# Cu-Enabled Photoinduced Charge Transfer in Green-Processed Cu-BPDC Metal–Organic Framework Films on FTO

**DOI:** 10.1021/acs.jpcc.6c04739

**Published:** 2026-09-25

**Authors:** Md Zahidul Hasan, Gan Xu, Kuo-Hao Chen, Angira Roy, Helen Qin, Shengjia Xiang, Sarah Yang, Yangchuan Xing, Gary Baker, Zhongyu Yang

**Affiliations:** † Department of Chemical and Biomedical Engineering, 3323 University of Missouri-Columbia, Columbia, Missouri 65211, United States; ‡ Department of Chemistry, 14716 University of Missouri-Columbia, Columbia, Missouri 65211, United States; § Department of Chemistry, 1259 University of Michigan, Ann Arbor, Michigan 48109, United States; ∥ Rock Bridge High School, Columbia, Missouri 65203, United States; ⊥ Materials Science & Engineering Institute, University of Missouri-Columbia, Columbia, Missouri 65211, United States

## Abstract

Photosensitive metal–organic frameworks (MOFs) provide chemically tunable platforms for studying light-induced charge transfer at solid/electrolyte interfaces, yet many reported systems rely on energy-intensive synthesis, organic solvents, or rare and/or toxic metal ions. Here, building on our previously reported ambient, water-assisted eco-LAGent synthesis of Cu-BPDC and Zn-BPDC MOFs, we formulate these materials as Nafion-bound films on fluorine-doped tin oxide (FTO) electrodes and evaluate their photoelectrochemical behavior. The resulting electrode platform enables a controlled comparison of the two MOFs under matched coating, electrolyte, and illumination conditions. The combination of earth-abundant Cu and Zn with the carboxylate ligand biphenyl-4,4′-dicarboxylate (BPDC) provides a chemically matched platform for comparing a redox-active Cu-BPDC MOF with a closed-shell Zn analogue under the same coating, electrolyte, and illumination geometry. Comparable scan-rate cyclic voltammetry (CV) showed a broad Cu-associated redox feature for Cu-BPDC, whereas Zn-BPDC remained comparatively featureless over the same potential window. Under chopped-light chronoamperometry (CA) at +0.449 V versus RHE, Cu-BPDC generated a sustained cathodic response of +0.448 ± 0.009 μA cm^–2^ when plotted as −Δj, while Zn-BPDC, bare FTO, and Nafion/FTO controls showed only weak responses under identical conditions. Optical measurements showed similar apparent direct-transition band gaps for Zn-BPDC and Cu-BPDC (∼4.00 eV), whereas distinct solid-state photoluminescence (PL) profiles indicated that optical absorption alone does not account for the different photoelectrochemical behaviors. These results support a Cu-centered photoinduced charge-transfer response in Cu-BPDC films and establish a controlled platform for interrogating metal-node-dependent photoelectrochemistry in MOF-based electrode coatings.

## Graphical abstract

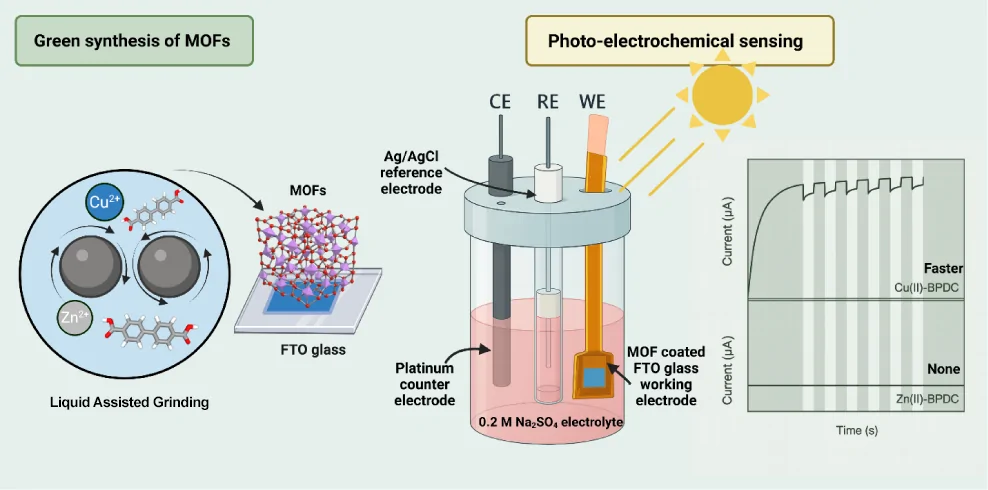

## 1 Introduction

Light-responsive materials that convert optical excitation into measurable charge-transfer events are central to photoelectrochemical sensing, solar-energy conversion, and photocatalytic/photovoltaic materials design.^1–8^ Among the current light sensitive materials,^9–13^ metal–organic frameworks (MOFs) are attractive because their metal nodes, organic linkers, pore environments, and film morphologies can be chemically tuned.^2,14–19^ In principle, these features can allow photoactivity to be designed and even tuned through the linker, the metal center, or the metal-linker interface. Some MOFs with larger pores have also been loaded with redox enzymes, enabling photoinduced biocatalytic reactions to be promoted (with the help of mediators in certain cases),^20^ further broadening the application of photosensitive MOFs. In practice, however, transferring MOF photophysics/photochemistry into electrode-level function requires not only a photoactive framework but also a reproducible, low-cost route that integrate MOFs with a conductive substrate to ensure electron flow through electrodes.

A second challenge is sustainability. Many crystalline MOF syntheses use high temperature, high pressure, long reaction times, or organic solvents, and some photoactive materials rely on highly toxic metal ions such as Cd^2+^, Cr^3+^, and Ni^2+^ that are undesirable for large-scale or environmentally exposed applications.^21–23^ The availability of certain metals is also limited on Earth, raising further questions about the resource scarcity and long-term scalability.^24–28^ To produce MOF photoelectrodes as green and sustainable materials, the synthesis and processing should be made milder while maintaining enough structural and electrochemical definition to support meaningful comparisons. Ambient aqueous or water-assisted approaches are, therefore, attractive synthesis routes in this context, particularly when combined with abundant metal salts and simple carboxylate linkers.

Classic aqueous phase MOF synthesis approaches relies on a cocrystallization or biomimetic mineralization,^29–33^ which would also allow the inclusion of redox enzymes for redox enzyme@MOF biocomposites as bioactive photosensitive materials. The limitation is the potential loss in crystallinity and poor yield for MOF crystals formed in water under ambient conditions. Liquid assisted grinding (LAG) or mechanic synthesis may overcome the low yield and crystallinity.^34–38^ However, most reported LAG synthesis of MOFs relies on organic solvents with only recent advances proving fully water-phase LAG synthesis for Zeolitic Imidazolate Frameworks (ZIFs).^39^ Recently, reported a water-assisted eco-LAGent approach for preparing a library of MOFs under mild conditions.^40^

Biphenyl-4,4′-prime-dicarboxylate (BPDC) is one of the carboxylate ligands we employed and a useful linker for exploring such systems because its aromatic structure can contribute to high-energy optical absorption while coordinating multiple divalent metal ions. What is unclear, however, is whether optical absorption by a common linker is sufficient to produce electrode-level photoresponse, or whether the metal node controls the redox-active pathway. Answering this question requires a comparison of MOFs prepared with BPDC with metal ions with distinct properties. Practically, a closed-shell Zn-BPDC and Cu-BPDC as an analogue could be candidate MOFs as the two materials share the BPDC linker but differ strongly in accessible metal-centered redox chemistry.

Building on our previously reported eco-LAGent synthesis of Cu-BPDC and Zn-BPDC MOFs,^40^ the main advance of the present work is their formulation as Nafion-bound films on fluorine-doped tin oxide (FTO) electrodes and their photoelectrochemical evaluation. We then compare their redox behavior and chopped-light photoelectrochemical response against bare FTO and Nafion/FTO controls under common cell geometry. Our data show that Cu-BPDC, but not Zn-BPDC or the substrate/binder controls, produces a sustained cathodic response under fixed-bias illumination. Optical band gap and photoluminescence measurements further show that the Cu/Zn-BPDC performance difference cannot be explained simply by the BPDC absorption edge, suggesting a metal-node-dependent photoinduced charge-transfer process. These findings establish Cu-BPDC as a green MOF coating capable of converting light exposure into a measurable electrochemical response under mild conditions. Beyond the specific Cu-BPDC/FTO system, our work suggests a broader design strategy in which green MOF synthesis, earth-abundant metal nodes, and processable thin-film formulations can be combined to develop photoresponsive materials for sensing, photocatalytic interfaces, and solar-driven electrochemical platforms. Our proof-of-principle data set thus, provides a foundation for future tuning of MOF composition, film thickness, binder chemistry, and electrode architecture.

## 2 Materials and Methods

Full experimental details, RHE conversion convention, illumination conditions, and electrochemical tests are provided in the Supporting Information (SI). Key procedures and experimental details such as electrode area, coating mass, and control geometry are summarized below as they are central to the interpretation of our data.

### 2.1 Eco-LAGent Synthesis of Cu-BPDC and Zn-BPDC

Cu-BPDC and Zn-BPDC MOFs were synthesized using an aqueous eco-LAGent method adapted from a previously reported protocol.^40^ Briefly, BPDC (∼77 mg) was combined with stainless-steel grinding beads in a short glass vial. Deionized water (50 μL) and triethylamine (88.2 μL) were added to promote ligand deprotonation, and the sealed vial was rolled at room temperature until a gel-like ligand mixture formed. The corresponding metal nitrate solution, Cu­(NO_3_)_2_ or Zn­(NO_3_)_2_, was then added and the mixture was rolled overnight to complete MOF formation. The solids were recovered, washed with water and methanol, dried under vacuum, and stored as dry powders before electrode ink preparation.

### 2.2 Preparation of MOF-Coated FTO Electrodes

FTO substrates were rinsed and sonicated in deionized water and ethanol, dried, and masked with Kapton tape to define a 0.5 cm × 0.5 cm exposed area. MOF coating suspensions were prepared by dispersing 3.00 mg MOF powder in 500 uL ethanol followed by addition of 20 uL Nafion dispersion. Films were prepared by sequential drop-casting 10 uL aliquots onto the exposed FTO area at 40 °C, with drying between layers. Ten coating cycles deposited an estimated 0.577 mg of MOF-containing suspension on the exposed window, corresponding to an areal loading of 2.31 mg cm^–2^. Sequential deposition with intermediate drying was used to reduce local pooling and improve reproducibility across the defined electrode area.

### 2.3 Photoelectrochemical Measurements

Electrochemical measurements were performed in 0.2 M Na_2_SO_4_ using the MOF-coated FTO as the working electrode, a Pt wire counter electrode, and an Ag/AgCl (sat. KCl) reference electrode. Potentials were converted to RHE using the convention described in the SI. The electrolyte was N_2_-purged before measurement and continuously bubbled with N_2_ during testing. Current densities were normalized to the 0.25 cm^2^ exposed electrode area. Illuminated measurements used an OAI TriSOL solar simulator (model 0131–0111–01; nominal lamp electrical power rating, 1000 W) providing broadband simulated solar illumination and positioned approximately 20 cm from the working electrode. Because irradiance at the working-electrode plane was not calibrated and *iR* compensation was not applied, the results are interpreted as relative dark/light comparisons under a common geometry rather than absolute photoelectrochemical performance metrics.

### 2.4 Optical Characterization

UV–vis absorption spectra were collected from ethanolic MOF-Nafion dispersions using Nafion in ethanol as the corresponding blank. Apparent optical band gaps were estimated from direct-transition Tauc plots by linear fitting of the absorption onset. Steady-state photoluminescence spectra were collected from MOF-Nafion thin films deposited on FTO. Spectra were corrected for instrument response and blank contributions as described in the SI. Photoluminescence excitation spectra and time-resolved PL decays were also collected for BPDC, Zn-BPDC, and Cu-BPDC, with detailed measurement and fitting procedures provided in the Supporting Information.

## 3 Results and Discussion

### 3.1 Green Synthesis and Film Fabrication of BPDC-Based MOF Photoelectrodes

The key to our materials design in this study is to prepare photosensitive MOF films on conductive electrode under mild materials-processing conditions, while retaining a well-defined electrode geometry for controlled photoelectrochemical comparison. Cu-BPDC and Zn-BPDC were synthesized under ambient conditions using a water-assisted eco-LAGent method, purified by washing with water and ethanol, and dried in oven before film fabrication as described before.^40^ This route bypasses the conventional solvothermal MOF synthesis and provides a sustainable preparation strategy for BPDC-based MOF crystals in high yield. The resulting powders were obtained in sufficient quantities for electrode fabrication and characterization, with approximately ∼30 mg of Cu-BPDC and Zn-BPDC per batch of LAG synthesis recovered from representative syntheses. Note that the eco-LAGent route does not guarantee intrinsically better photoelectrochemical performance than solvothermally prepared MOFs; instead, our goal is to test whether a mildly prepared MOF can be directly translated into a functional electrode coating. Its main advantage is therefore reduced synthesis severity, including ambient processing and reduced use of organic solvent during MOF formation, while retaining sufficient material quality for electrode fabrication and comparative photoelectrochemical measurements.

To convert these MOF powders into processable photoelectrode coatings, each MOF was dispersed in ethanol containing Nafion binder and mechanically deposited onto masked FTO electrodes by sequential drop-casting (Scheme 1). The same drop-casting protocol was used in terms of dispersion concentration, deposition volume, number of coating steps, drying conditions, and exposed electrode area for all MOF-containing samples. Specifically, our MOF dispersion concentration is ∼6.00 mg mL^–1^, Nafion content is ∼20 μL, total drop-cast volume is 500 μL and estimated MOF loading is ∼2.31 mg cm^–2^. This produced visually continuous MOF/Nafion films over a defined 0.25 cm^2^ window, allowing currents to be normalized to the same geometric electrode area. Note that it is critical to use identical substrate, binder, loading protocol, and exposed area because it would allow the subsequent electrochemical and chopped-light responses to be compared as a function of MOF composition rather than differences in electrode geometry or film preparation.

**Scheme 1:**
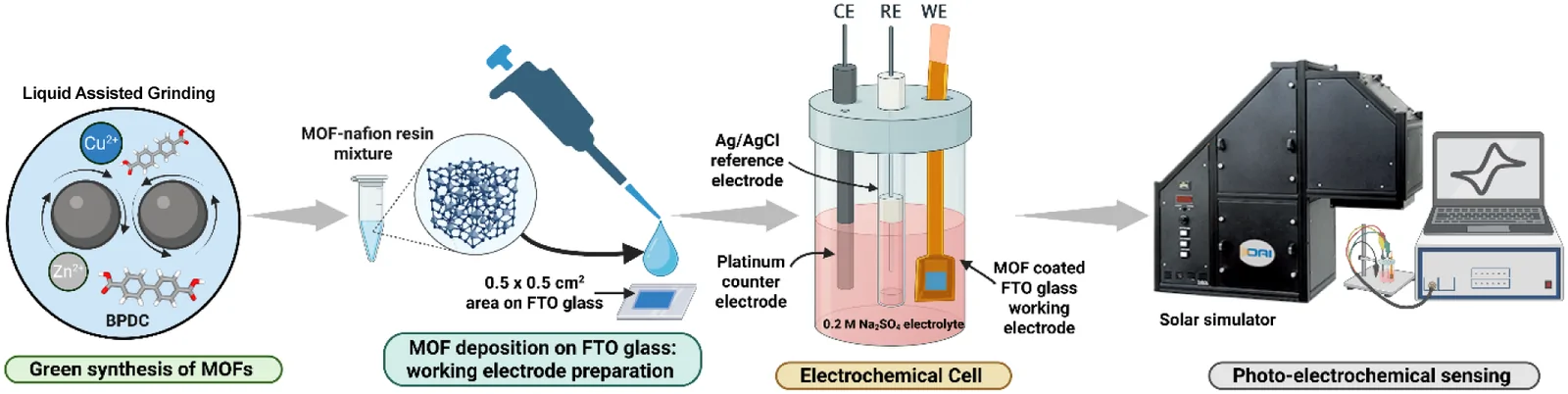
Schematic Illustration of the Preparation of MOF-Coated FTO Electrodes and Photoelectrochemical Sensing under Simulated Solar Light

We then carried out basic characterization confirmed that the ambient eco-LAGent route produced BPDC-based MOF solids suitable for comparative electrode testing (Figure 1). Specifically, the powder X-ray diffraction (PXRD) patterns showed diffraction features consistent with BPDC-based Cu and Zn MOFs prepared in our previous work and related reports (Figure 1a & 1d black).^40,41^ Scanning electron microscope (SEM) further confirmed the formation of particulate MOF solids with distinguishable morphologies for the Cu-BPDC and Zn-BPDC samples (Figure 1b & 1e). Comparing to our recently published characterization of Cu-BPDC and Zn-BPDC MOFs synthesized by LAG, the consistency with known BPDC-based MOF supports the successful formation of BPDC-based MOF materials (note that there is no need for a full crystallographic refinement or definitive phase assignment to confirm the formation of the needed MOFs). PXRD patterns collected before and after light exposure retained the characteristic diffraction features of both Cu-BPDC and Zn-BPDC, indicating no obvious gross structural change under the tested illumination conditions (Figure 1a,d). The corresponding SEM images qualitatively show retention of the particulate morphology (Figure 1b, c, e, f), although they are not used here to make a quantitative before/after morphological comparison. Post-testing SEM-EDS further confirmed retention of Cu- and Zn-containing material on the electrodes, although Cu-BPDC showed greater compositional redistribution after photoelectrochemical testing (Figures S1–S4). Together with additional characterization (Figure s S5–S6), our results establish that Cu-BPDC and Zn-BPDC can be prepared by a mild, water-assisted route and processed into comparable FTO-supported MOF films. This materials-level control is essential for the following electrochemical analysis: because the Cu and Zn samples share the same BPDC linker, electrode substrate, binder, coating protocol, and testing geometry, differences in redox and photoelectron-chemical behavior can be evaluated primarily by the metal node identity.

**Figure 1:**
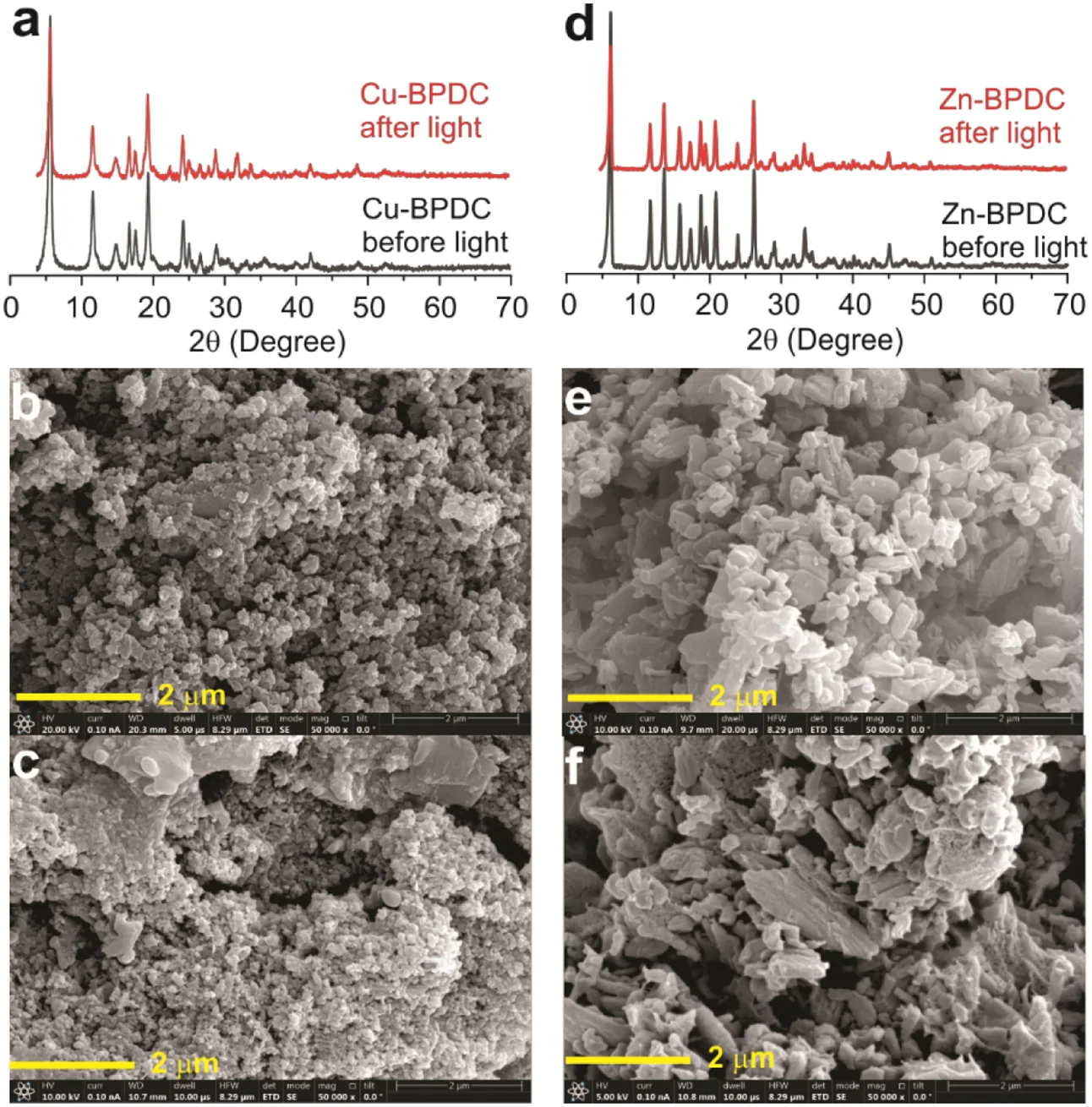
PXRD and SEM characterization of Cu-BPDC and Zn-BPDC before and after light exposure. (a, d) PXRD patterns of Cu-BPDC and Zn-BPDC before and after illumination. (b, c) Representative SEM images of Cu-BPDC before and after illumination, respectively. (e, f) Representative SEM images of Zn-BPDC before and after illumination, respectively.

### 3.2 Cu-BPDC Displays a Film-Coupled Redox Response Absent from Zn-BPDC

After establishing MOF-coated FTO electrodes, we then used scan-rate-dependent cyclic voltammetry (CV) to determine whether the Cu-BPDC and Zn-BPDC films contained any electrochemical redox sites under the electrolyte and potential conditions used for typical photoelectrochemical testing (Figure 2). We found that Cu-BPDC exhibited a broad cathodic feature within the measured potential window, whereas Zn-BPDC showed only weak, featureless current over the same scan-rate range. For Cu-BPDC, the apparent cathodic peak shifted from +0.517 to +0.456 V versus RHE as the scan rate increased from 25 to 200 mV s^–1^, while the cathodic current-density magnitude increased from 132 to 192 μA cm^–2^ (Table S1). The consistency of this feature across different scan rates suggested Cu-BPDC to be an electrochemically active film, while the peak shift and broad line shape indicated that the response is not an ideally reversible solution-like Cu couple.

**Figure 2:**
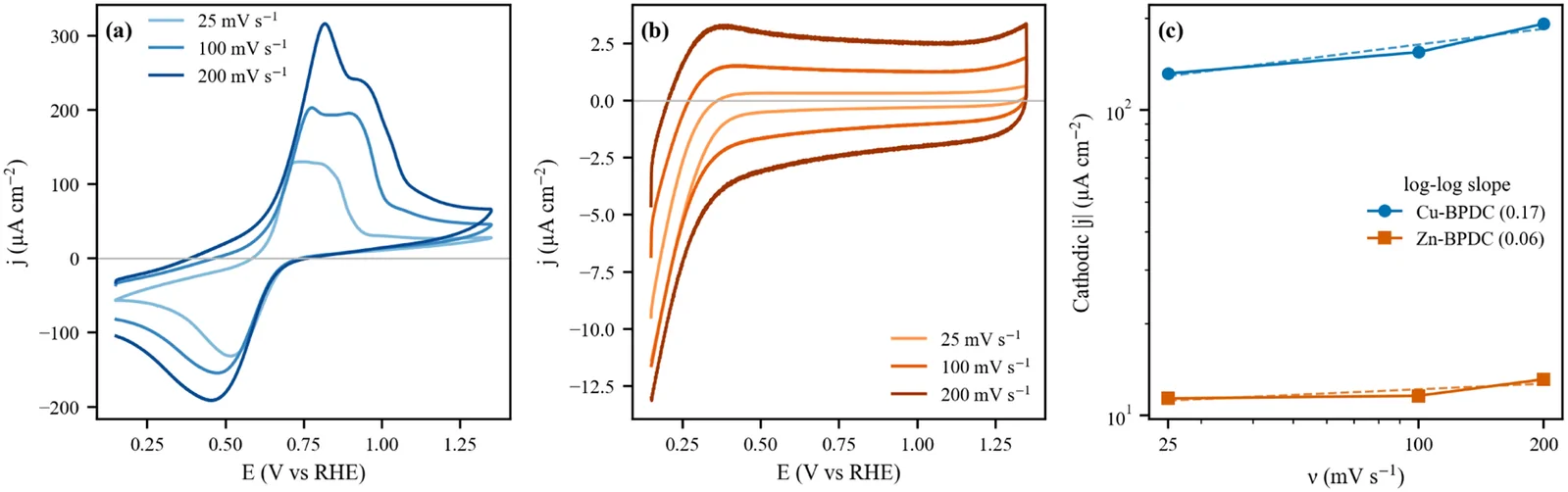
Last-cycle CV comparison of Cu-BPDC and Zn-BPDC at matched scan rates of 25, 100, and 200 mV s^–1^. (a) Cu-BPDC shows a broad redox feature within the measured potential window. (b) Zn-BPDC remains relatively featureless under the same conditions. (c) Cathodic current-density values extracted from the Cu-BPDC and Zn-BPDC traces as a function of scan rate.

The scan-rate dependence provides additional insight into the nature of the Cu-BPDC response. The negative shift of the cathodic peak with increasing scan rate suggests kinetic polarization, ion-transport limitations, or film-coupled charge compensation during reduction of Cu sites within the MOF/Nafion coating. The broad peak shape and anodic/cathodic asymmetry are consistent with a heterogeneous film environment in which Cu centers are distributed across different local coordination, electronic, or interfacial environments rather than behaving as a single freely diffusing redox species. Therefore, the Cu-BPDC voltammetric feature is best interpreted as a film-associated Cu redox fingerprint (rather than a fully reversible molecular redox couple).

Zn-BPDC served as an important closed-shell comparison. Because Zn^2+^ is not expected to undergo accessible Zn-centered redox chemistry within this potential window, the nearly featureless Zn-BPDC voltammograms indicate that the BPDC linker, Nafion binder, and FTO substrate do not by themselves display/possess the broad redox response observed for Cu-BPDC. This distinction is essential for the later interpretation of the light-response data: the two films share the same organic linker, substrate, binder, geometric area, and coating procedure, but only Cu-BPDC provides an electrochemically active metal node. The CV results therefore separate metal-centered redox activity from linker-associated optical absorption or nonspecific film capacitance.

A postseries CV check (Figure S7) further showed that the Cu-BPDC peak position remained within approximately 30 mV, whereas the apparent peak amplitude decreased substantially after repeated scans. This behavior indicates that the Cu-associated redox feature remains identifiable, while the electrochemically accessible response decreases during repeated cycling. Therefore, the peak position is used here as a diagnostic redox fingerprint, while the absolute peak amplitude is treated as history-dependent rather than an intrinsic material constant. Importantly, the fixed bias used for chopped-light chronoamperometry (CA) in the next section, +0.449 V versus RHE, lies near the Cu-BPDC redox region identified by CV. The following experiments therefore, test whether illumination produces a measurable current response specifically at a redox-active Cu-BPDC interface, rather than at a redox-silent BPDC film or the underlying FTO/Nafion controls.

Post-testing SEM-Energy Dispersive X-ray Spectroscopy (EDS) provided additional information on changes in the electrode coating during photoelectrochemical testing (Figures S1–S4). Cu remained broadly distributed across the Cu-BPDC electrode after photoelectrochemical testing, indicating that Cu-containing material was retained on the electrode. However, the normalized Cu contribution decreased while the Si contribution from the underlying substrate increased, consistent with some degree of coating redistribution, thinning, cracking, and/or partial material loss. In contrast, the Zn-BPDC electrode showed comparatively smaller compositional changes, with broadly distributed Zn retained after testing. Because SEM-EDS cannot distinguish among physical loss, changes in electrical contact, framework dissolution, or electrochemical reconstruction, we do not assign the decrease in Cu-BPDC CV amplitude to a specific degradation mechanism.

### 3.3 Chopped-Light Chronoamperometry (CA) Identifies a Fixed-Bias Cathodic Response Specific to Cu-BPDC

Chopped-light CA was used as the primary test for photoelectrochemical response because it isolates the current change caused by illumination at a fixed applied bias potential. It is complementary to CV in a way that CV establishes whether the film contains an electrochemically detectable redox active site, whereas chopped-light CA tests whether illumination perturbs the current at a selected potential within that electrochemical window. Chopped-light CA was measured for Cu-BPDC and Zn-BPDC, together with negative controls such as bare FTO, and Nafion/FTO electrodes +0.449 V versus RHE using 100 s dark/light intervals (Figure 3). The +0.449 V vs RHE bias was selected because it lies near the cathodic Cu-BPDC redox region identified by CV (approximately +0.456 to +0.517 V vs RHE over the scan rates examined), allowing the light response to be evaluated under conditions where the Cu-containing film is electrochemically active. Because the first light-on interval was dominated by baseline relaxation, it was excluded from quantitative comparison and only cycles 2–5 were used for the reported light-dark response.

**Figure 3:**
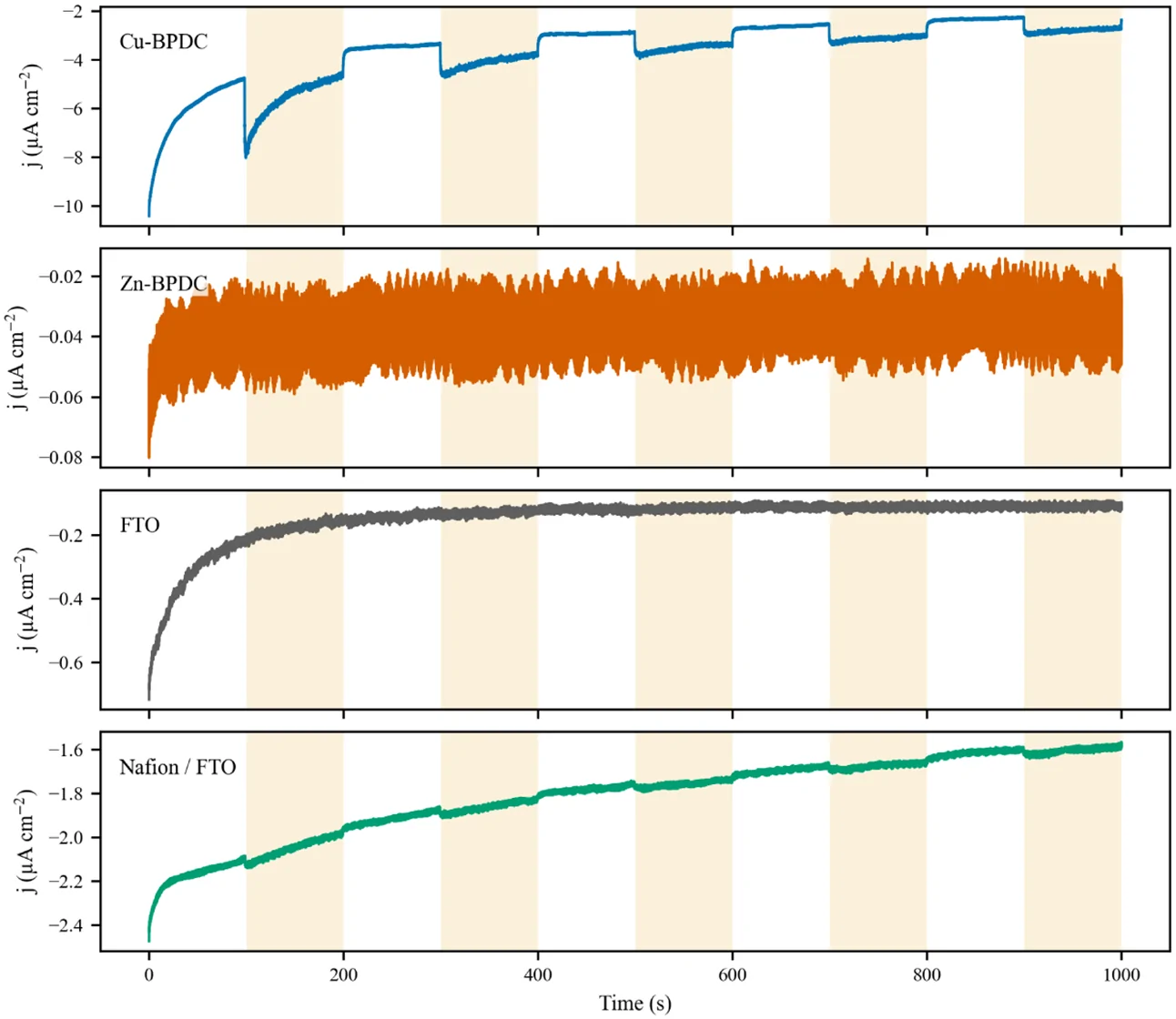
Chopped-light CA current–time traces collected at +0.449 V versus RHE (−0.20 V versus Ag/AgCl) with 100 s dark/light intervals. Shaded regions denote illumination. The first light-on period includes baseline relaxation; the sustained light–dark response was quantified from cycles 2–5, as summarized in Figure 3.

For Cu-BPDC, illumination shifted the current in the cathodic direction. Quantitatively, the Cu-BPDC response in Δj was −0.448 ± 0.009 μA cm^–2^, or +0.448 ± 0.009 μA cm^–2^ when plotted as the cathodic response −Δj (Figure 4). The corresponding responses of Zn-BPDC, bare FTO, and Nafion/FTO were near zero or opposite in sign under the same analysis convention. These controls separate the Cu-BPDC response from possible artifacts. Specifically, the bare FTO tests whether the conductive substrate itself generates a chopped-light current, the Nafion/FTO tests whether the binder or film-forming layer contributes to the response, and Zn-BPDC tests whether a BPDC-containing MOF film with similar processing but without an accessible Cu redox center is sufficient to produce the same photocurrent. The absence of a comparable cathodic response in all 3 controls indicates that the measured fixed-bias response is not simply caused by the FTO substrate, Nafion binder, BPDC linker absorption, or nonspecific illumination of the electrochemical cell.

**Figure 4:**
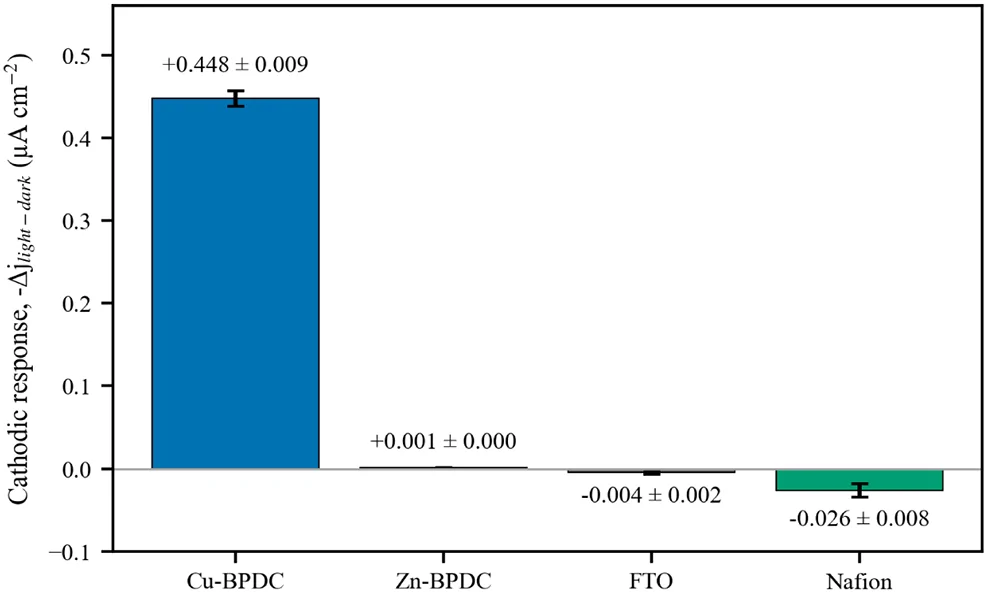
Quantified chopped-light CA response from cycles 2–5. Bars show the cathodic response, calculated as −Δj, where Δj = j_light_ – j_dark_. Under this sign convention, a positive value means that illumination makes the current more cathodic. Error bars are the standard deviation of four light/dark cycles within the same CA measurement and electrode.

The sign of the Cu-BPDC response is also informative. At the applied bias potential, illumination makes the current more cathodic, indicating that light promotes a net reduction-direction current or shifts the interfacial charge-transfer balance toward electron-consuming processes. Because Zn-BPDC does not show a similar behavior, such response cannot be assigned only to high-energy absorption by the shared BPDC linker. Instead, the CA result is most consistent with a Cu-associated, photoinduced charge-transfer process in which optical excitation changes the population, accessibility, or interfacial electron-transfer behavior of Cu redox-active sites within the MOF/Nafion film. Our interpretation remains intentionally conservative though; the CA experiment identifies a Cu-specific light-induced electrochemical response, but it does not by itself prove a single microscopic pathway such as ligand-to-metal charge transfer.

The dark/light linear sweep voltammetry (LSV) measurements (Figure S8) further emphasize that the Cu-BPDC photoresponse is not a simple mode-independent baseline shift. During slow-sweep LSV, illumination reduced the magnitude of the cathodic Cu-BPDC redox peak, whereas fixed-bias CA at +0.449 V versus RHE produced a sustained shift toward more cathodic current. This difference suggests that illumination affects the film differently under dynamic potential scanning and steady applied bias potential. Under LSV conditions, the measured response reflects how light alters the evolving Cu redox feature across a changing potential window. Under CA conditions, the electrode is held near the Cu-BPDC redox region, so the observed current reflects the steady light-induced perturbation of the film/electrolyte interface. The opposite LSV and CA signs therefore, should not be treated as a contradiction; rather, they indicated that the photoelectrochemical response depends on the electrochemical operating mode.

The Cu-BPDC light-on transients (Figure S9) gave an apparent switching time of 316 ± 77 ms from 4 illumination cycles. This subsecond response supports a prompt light-induced current change in the Cu-BPDC film coated on our electrode within one CA measurement and the presence of residual baseline relaxation. Together with the CV results, the chopped-light CA data suggested Cu-BPDC as the only film in the control set that combines an accessible redox feature with a sustained cathodic response under fixed-bias illumination. This result provides the central photoelectrochemical evidence for a Cu-centered light-responsive MOF coating and motivates some optical analysis, where absorption-edge and photoluminescence data can test whether the Cu/Zn difference can be explained by optical absorption alone.

### 3.4 Optical Measurements Separate Shared BPDC Excitation from Metal-Dependent Photoelectrochemical Behavior

Optical measurements were used to test whether the different photoelectrochemical responses of Cu-BPDC and Zn-BPDC could be explained simply by different light-absorption edges. UV–vis/Tauc analysis was performed on MOF–Nafion dispersions using Nafion in ethanol as the blank (Figure 5). Direct-transition Tauc plots indicated apparent optical band gaps of 4.01 eV for Zn-BPDC and 4.00 eV for Cu-BPDC which are essentially indistinguishable under the present testing conditions. For comparison, application of the same Tauc analysis to the free Na_2_BPDC linker yielded an apparent optical transition energy of 4.07 eV (Figure S10). Thus, both frameworks exhibit apparent absorption edges within ∼0.06–0.07 eV of the free linker and within 0.01 eV of one another. This close correspondence is consistent with a substantial contribution from the shared BPDC chromophore to the high-energy optical onset, although the Tauc-derived values do not establish identical electronic structures.

**Figure 5:**
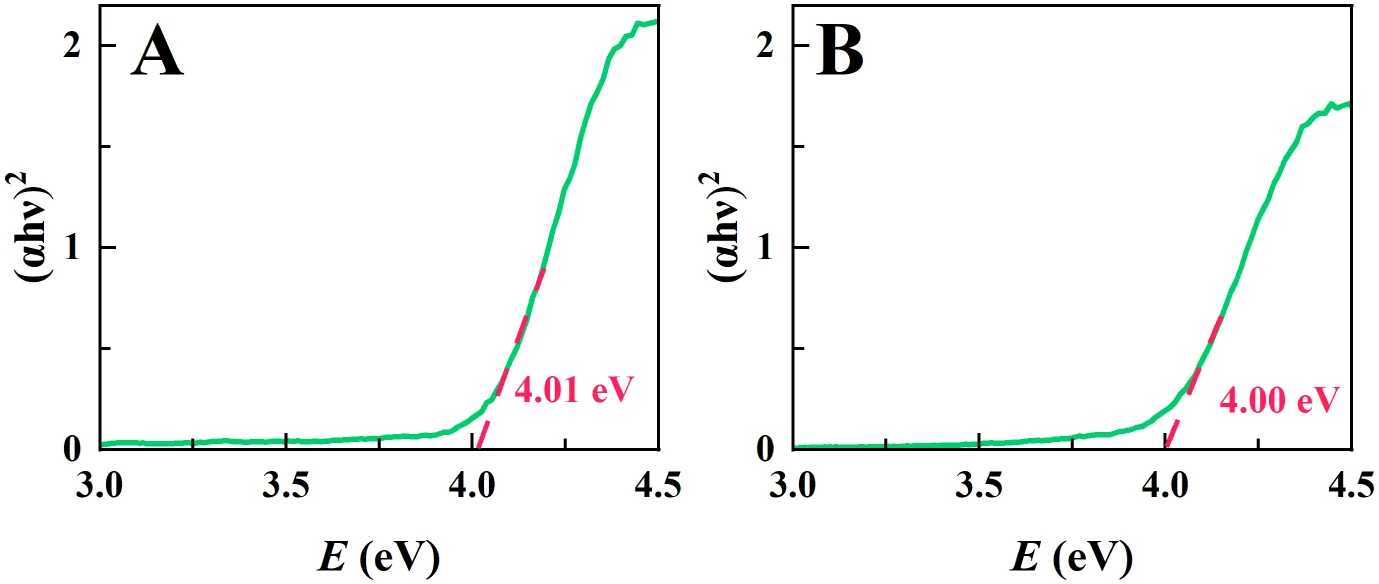
Direct-transition Tauc plots of ethanolic MOF dispersions of (A) Zn­(II)-BPDC and (B) Cu­(II)-BPDC were used to estimate their optical band gap energies, *E*_g_. Plots of (αhν)^2^ versus photon energy (hν) are shown, where α is the absorption coefficient, yielding equivalent values of ∼4.00 eV for both MOFs.

This finding argues against the simplest explanation for the electrochemical contrast. If Cu-BPDC produced a stronger chopped-light response merely because it had a substantially lower-energy absorption edge than Zn-BPDC, the Tauc analysis would show a clear difference between the two films. Instead, both BPDC frameworks exhibit similar apparent band gap energies. Therefore, the Cu-specific cathodic response observed by CA cannot be attributed only to the presence of the BPDC linker or to a gross difference in absorption-edge position. Rather, the optical and electrochemical data together suggest that absorption by the BPDC-based framework must be coupled to an additional metal-dependent pathway that is available in Cu-BPDC but not in the closed-shell Zn analogue.

Solid-state photoluminescence (PL) measurements provided a complementary comparison of excited-state relaxation in the MOF-containing films. Nafion alone showed only weak emission centered near approximately 335 nm, whereas both MOF-containing films exhibited stronger, red-shifted emission with maxima near 400 nm (Figure S11). The red-shifted emission relative to Nafion supports the assignment that the observed PL response arises primarily from the MOF component rather than from the binder or FTO-supported Nafion matrix. Normalized PL spectra (Figures S12–S15) further showed reproducible differences between Zn-BPDC and Cu-BPDC, with Zn-BPDC displaying a broader long-wavelength tail under the tested conditions.

To further evaluate the excited-state behavior, photoluminescence excitation (PLE) and time-resolved PL measurements were performed for free BPDC, Zn-BPDC, and Cu-BPDC. The three materials exhibited similar PLE profiles with excitation maxima near 340 nm (Figures S12–S15), providing complementary evidence that the emissive states of both frameworks are accessed through a common BPDC-centered excitation (Figure S16). Time-correlated single-photon counting measurements showed that the intensity-weighted average PL lifetime decreased from 38.7 ns for free BPDC to 7.3 ns for Zn-BPDC and 9.2 ns for Cu-BPDC (Figure 6 and Table S2). These results show that framework formation introduces additional excited-state deactivation pathways, while the similar lifetimes of Zn-BPDC and Cu-BPDC indicate that the relaxation behavior probed by PL does not differ dramatically between the two frameworks. Importantly, these measurements do not distinguish among nonradiative relaxation, energy transfer, charge transfer, or other framework-mediated processes and therefore do not establish a specific microscopic charge-transfer mechanism. Rather, the optical data show that the distinct Cu-BPDC photoelectrochemical response occurs despite broadly similar BPDC-centered excitation and excited-state lifetimes, supporting a role for metal-node identity in the photoelectrochemical response. Together, the optical measurements show that Cu-BPDC and Zn-BPDC have similar apparent absorption-edge energies, similar BPDC-centered excitation profiles, and broadly similar excited-state lifetimes, despite their very different electrochemical light responses. Thus, the optical data do not identify the microscopic origin of the photocurrent, but they indicate that the Cu/Zn photoelectrochemical contrast cannot be explained simply by different optical excitation thresholds or by BPDC photophysics alone.

**Figure 6:**
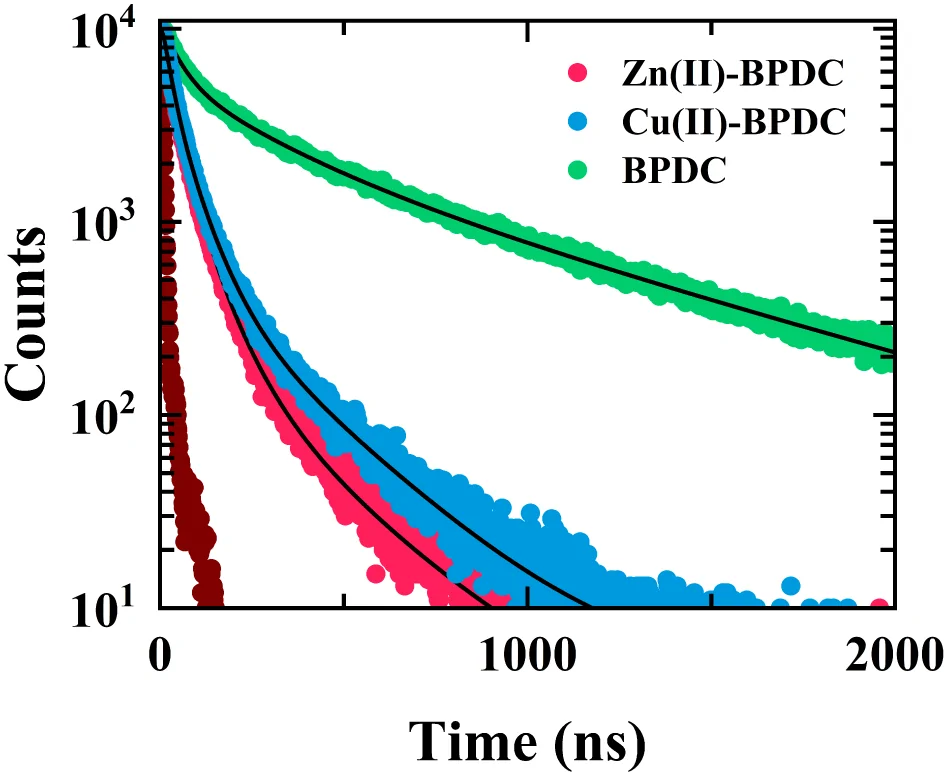
Time-resolved PL decays of drop-cast BPDC, Zn­(II)-BPDC, and Cu­(II)-BPDC films on FTO recorded by TCSPC (λ_ex_ = 290 nm), collected at the respective emission maxima. Solid black lines are triexponential fits; the corresponding fit parameters and intensity-weighted average lifetimes are given in Table S4. The dark red trace shows the instrument response function.

### 3.5 Mechanistic Interpretation, Scope, and Design Implications

The materials, electrochemical, and optical results establish a Cu-dependent photoelectrochemical response in Cu-BPDC films and are consistent with involvement of the Cu node in the interfacial charge-transfer process. The ambient eco-LAGent synthesis and matched FTO coating protocol establish Cu-BPDC and Zn-BPDC as a controlled BPDC-based materials pair. The CV data show that only Cu-BPDC contains an electrochemically detectable redox feature in the tested potential window, whereas Zn-BPDC remains relatively featureless. The chopped-light CA results then show that only Cu-BPDC produces a sustained cathodic response at fixed bias, while Zn-BPDC, bare FTO, and Nafion/FTO show much weaker or oppositely signed responses. Finally, the optical data show that Cu-BPDC and Zn-BPDC have nearly identical apparent absorption-edge energies but different photoluminescence profiles. This combination of evidence argues against assigning the photoresponse solely to BPDC absorption, the FTO substrate, or the Nafion binder.

One plausible interpretation is that the BPDC framework contributes high-energy optical absorption, while the Cu centers provide an accessible redox pathway that allows illumination to perturb interfacial charge transfer under fixed-bias conditions. In this view, the Cu node is not just a structural component of the MOF but an active participant in the light-responsive electrochemical behavior. Zn-BPDC is valuable because it separates the role of the shared linker from the role of the redox-active metal node. Despite having a similar apparent optical band gap, Zn-BPDC lacks the Cu-associated redox feature and does not produce a comparable cathodic chopped-light response.

The present results support a Cu-associated photoelectrochemical response, but they do not by themselves prove a single microscopic charge-transfer pathway such as ligand-to-metal charge transfer (LMCT). Direct assignment of LMCT, metal-centered excited states, or defect-mediated pathways would require additional evidence, such as transient spectroscopy, operando spectro-electrochemistry, electronic-structure calculations, XPS/EPR before and after bias or illumination, or dark/light electrochemical impedance spectroscopy.

These limitations do not weaken the central materials conclusion; rather, they define the level at which the current study should be interpreted. The work establishes a mild-processing route for preparing BPDC-based MOF films and shows that replacing closed-shell Zn with redox-active Cu changes both the electrochemical and light-responsive behavior under otherwise matched conditions. This finding provides a practical design principle for sustainable MOF photoelectrodes: green synthesis and simple film processing can be combined with metal-node selection to tune interfacial photoelectrochemical response. Future optimization should focus on calibrated illumination, independent electrode replicates, film-thickness and loading control, binder/electrode architecture, and operando measurements that directly resolve the Cu-centered charge-transfer pathway. In general, open-shell or redox-active metal ions other than Cu may introduce additional metal-centered or metal-mediated electronic pathways, whereas closed-shell ions may provide useful controls; however, the resulting photoelectrochemical behavior cannot be predicted from d-electron configuration alone because it will also depend on oxidation state, coordination environment, MOF structure, and energetic alignment with the linker and electrode. Future studies comparing additional organic linkers and metal nodes under matched electrochemical and optical conditions will help separate the respective contributions of linker identity, metal-node redox properties, and metal-linker interactions to the observed photoresponse.

## 4 Conclusions

This work demonstrates the fabrication and photoelectrochemical evaluation of Nafion-bound Cu-BPDC and Zn-BPDC films on FTO using MOFs prepared by our previously reported mild, water-assisted eco-LAGent route. By comparing Cu-BPDC with the closed-shell Zn-BPDC analogue under the same coating protocol, electrolyte, illumination geometry, and geometric electrode area, the study separates metal-node-dependent photoelectrochemical behavior from nonspecific contributions of the BPDC linker, FTO substrate, and Nafion binder. Cu-BPDC exhibited a broad Cu-associated redox feature and generated a sustained cathodic response under chopped illumination at +0.449 V versus RHE, whereas Zn-BPDC, bare FTO, and Nafion/FTO showed only weak or oppositely signed responses. Optical measurements further showed nearly identical apparent direct-transition band gaps for Zn-BPDC and Cu-BPDC, but distinct photoluminescence profiles, indicating that the photoelectrochemical contrast cannot be explained by BPDC absorption alone. Optical measurements further showed nearly identical apparent direct-transition band gaps and similar BPDC-centered excitation behavior for Zn-BPDC and Cu-BPDC, indicating that the photoelectrochemical contrast cannot be explained by differences in optical excitation alone. These results establish Cu-BPDC as a green-processed MOF coating capable of producing a measurable Cu-dependent photoelectrochemical response. The microscopic origin of this response, however, remains unresolved. More broadly, the work provides a practical materials platform for designing sustainability-oriented MOF photoelectrodes in which mild synthesis, processable thin-film formulation, and redox-active metal-node selection are combined. Such platforms may be useful for future development of light-responsive electrochemical interfaces, photoelectrochemical sensing coatings, and model systems for studying charge transfer in MOF films. Future studies will be focused on operando spectroscopic or computational tests to confirm the proposed Cu-centered charge-transfer pathway. Future studies will focus on operando spectroscopic and computational approaches to investigate the microscopic origin of the observed Cu-dependent photoelectrochemical behavior. Future optimization could also include spray-based deposition and quantitative control of film thickness and coverage.

## Supplementary Material



## Notes

Resources of chemical supplies; details of MOF composite synthesis; basic characterization of the prepared composites; supporting characterization data; raw data from the enzyme activity assay; supporting tables of experimental observations and simulation parameters (PDF)
