# Supplementary material for: Cu-Enabled Photoinduced Charge Transfer in Green-Processed Cu-BPDC Metal–Organic Framework Films on FTO
Source: J Phys Chem C Nanomater Interfaces. 2026 Sep 25;130(40):14040–9. doi: 10.1021/acs.jpcc.6c04739 (PMC13656778; doi:10.1021/acs.jpcc.6c04739)
Supplement: Supplementary file 1 [file jp6c04739_si_001.pdf]

Supporting Information for

Cu-Enabled Photoinduced Charge Transfer in Green-Processed Cu-BPDC Metal-Organic Framework Films on FTO

Md Zahidul Hasan,[1] Gan Xu,[1] Kuo-Hao Chen,[1,*] Angira Roy,[2] Helen Qin,[3] Shengjia Xiang,[4] Sarah Yang,[4] Yangchuan Xing,[1] Gary Baker,[2,5,*] Zhongyu Yang[1,5,*]

1. Department of Chemical and Biomedical Engineering, University of Missouri-Columbia, Columbia, MO 65211

2. Department of Chemistry, University of Missouri-Columbia, Columbia, MO 65211

3. Department of Chemistry, University of Michigan, Ann Arbor, MI, 48109

4. Rock Bridge High School, Columbia, MO 65203

5. Materials Science & Engineering Institute, University of Missouri-Columbia, Columbia, MO 65211

Corresponding to: zhongyu.yang@missouri.edu

bakergar@missouri.edu

kchen39@ua.edu

Basic Materials

Zinc nitrate (Sigma-Aldrich no. 228737)

Copper nitrate (Chem Impex International Inc no. 30277)

4,4'-Biphenyldicarboxylic Acid (BPDC-COOH, Chem Impex International Inc no. 26841)

Nafion™ perfluorinated resin (Sigma-Aldrich no. 663492)

Fluorinated doped tin oxide glass slide (Sigma-Aldrich no. 735140)

Ethanol (95 %, standard resources from any commercial resources)

NaOH (standard resources from any commercial resources)

Isopropanol (standard resources from any commercial resources)

Triethylamine (standard resources from any commercial resources)

Double distilled (dd) water (standard resources)

Supporting Methods

Metal-Organic Framework (MOF) Formation:

Cu-BPDC and Zn-BPDC MOFs were synthesized using an aqueous eco-friendly liquid-assisted grinding (eco-LAGent) method adapted from a previously reported protocol.[1] Briefly, ~77 mg of biphenyl-4,4′-dicarboxylic acid (BPDC-COOH) was added to a 30 mL glass vial containing 6-8 stainless-steel grinding beads. 50 µL of deionized water and 88.2 µL of triethylamine were then added to the vial to promote ligand deprotonation. The sealed vial was then placed on a laboratory roller and mixed at 25-35 rpm for 90 min at room temperature, during which a thick, gel-like ligand mixture was formed. Next, 474 µL of 0.5 M metal nitrate solution, $Cu(NO_3)_2$ for Cu-BPDC or $Zn(NO_3)_2$ for Zn-BPDC, was added to the vial. The reaction mixture was sealed again and rolled overnight at room temperature under the same rolling rate.

The resulting MOF solids were recovered by adding 500 μL of deionized water to detach the product from the vial surface, followed by transferring the suspension into microcentrifuge tubes. All samples were centrifuged at 16,000 rpm for 1 min with the supernatant discarded. The collected pellets were washed 3-5 times, first with deionized water to remove unreacted metal ions and ligand residues, followed by methanol to remove residual water and loosely attached species. The purified Cu-BPDC and Zn-BPDC powders were dried under vacuum for around 24 hours to remove residual solvent and stored as dry powders until use.

MOFs coating on Fluorine-doped tin oxide (FTO):

FTO electrodes were cleaned with water and ethanol prior to coating to remove surface contaminants. The FTO substrates were then rinsed with deionized water and ethanol, followed by sequential sonication in deionized water and ethanol. The cleaned substrates were then dried before use. Only the FTO substrate containing side was recognized as the conductive side. The active electrode area was defined by masking the FTO surface with Kapton thin polyimide tape (Cat. No. 77708-07), leaving a geometric exposed area of 0.5×0.5 cm^2 for MOF deposition (Scheme S1a).

The MOF coating suspension was prepared by dispersing 3.00 mg of MOF in 500 μL of ethanol, followed by the addition of 20 μL of Nafion dispersion (1100 EW, 20 wt%; Ion Power, D2021) as a binder. The nominal MOF concentration was ~5.77 μg/μL.

MOF-coated FTO electrodes were prepared by sequential layer-by-layer drop-casting. During deposition, the FTO electrode was kept on a hotplate at 40 °C. The MOF suspension was deposited onto the exposed FTO area in sequential 10 μL aliquots. Each layer was dried for 4 min before deposition of the subsequent aliquot. The process was repeated for 10 coating cycles, resulting in a total deposited suspension volume of 100 μL. The total estimated MOF mass deposited onto the FTO electrode was ~0.577 mg, resulting in an areal loading of 2.31 mg/cm^2 (Scheme S1b-c).

Photo-Electrochemical Setup:

Electrochemical measurements were used to distinguish the Cu-BPDC photoresponse from the background responses of Zn-BPDC, bare FTO, and Nafion/FTO. Potentials are reported *versus* reversible hydrogen electrode (RHE) after conversion from an Ag/AgCl (sat. KCl) reference electrode (Scheme S1d). The electrolyte, 0.2 M Na_2SO_4, was N_2-purged before measurement and continuously bubbled with N_2 during testing. A 0.6 mm Pt wire was used as the counter electrode. Current densities were normalized to the 0.25 cm² exposed FTO window defined by the 0.5 cm × 0.5 cm opening in the 3D-printed cap, which also allowed direct illumination of the working electrode. Illuminated measurements were performed using the solar simulator OAI TriSOL, placed ~ 20 cm from the working electrode.

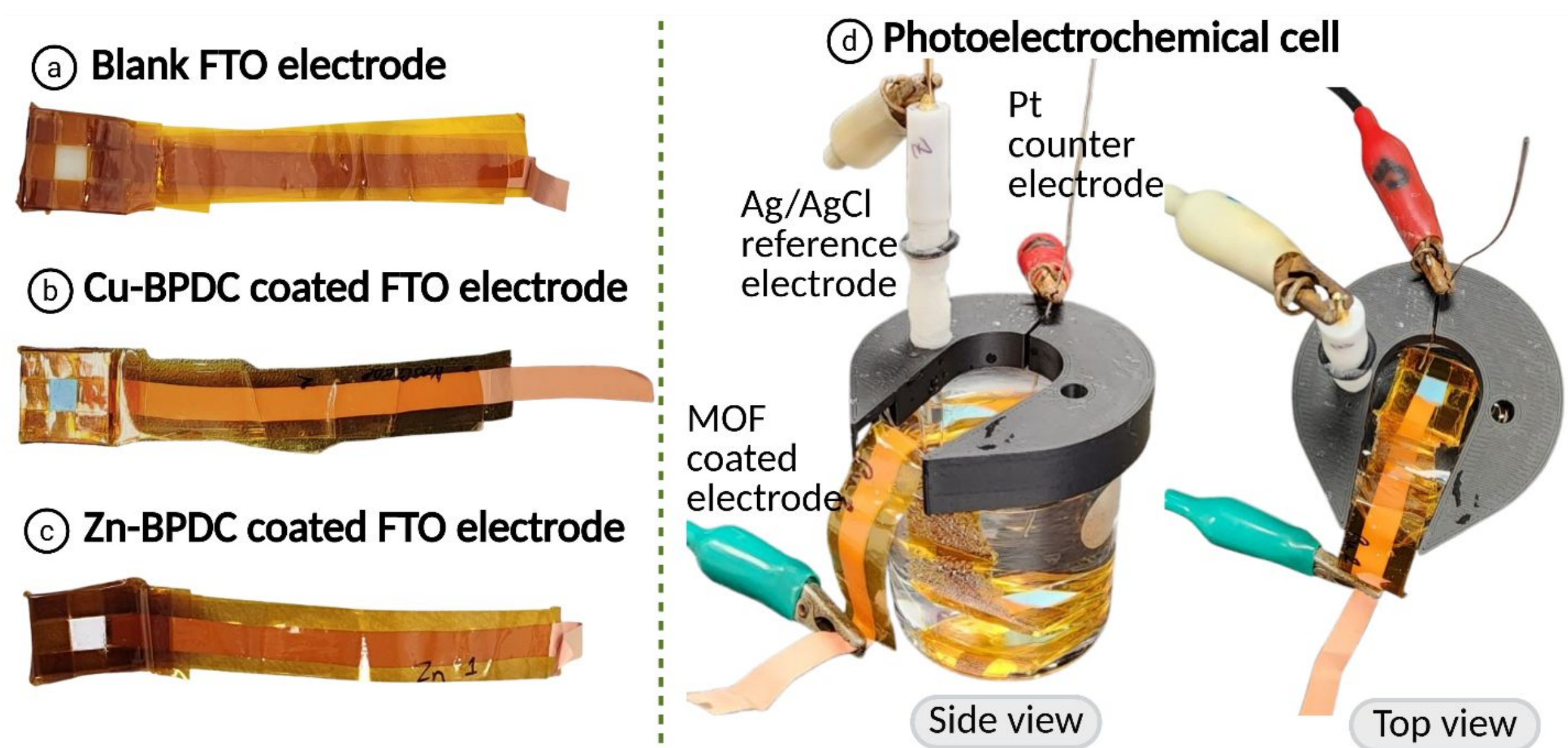

Scheme S1: MOF-coated FTO electrodes and the photoelectrochemical cell configuration. (a) Blank FTO electrode, (b) Cu-BPDC-coated FTO electrode, and (c) Zn-BPDC-coated FTO electrode, and (d) side and top views of the assembled three-electrode photoelectrochemical cell

containing the MOF-coated FTO working electrode, Ag/AgCl reference electrode, and Pt counter electrode.

Scanning Electron Microscope (SEM)

Typically, dried MOFs were attached on aluminum mounts using carbon adhesive tabs/tape and then coated with a conductive layer of carbon in a high-vacuum evaporative coater (Cressington 208c, Ted Pella Inc., Redding, California, USA). The morphology of the composites was acquired using a JEOL JSM-7600F scanning electron microscope (JEOL USA Inc., Peabody, Massachusetts) operating at 2 kV.

Powder X-ray Diffraction (PXRD)

The dried sample was loaded into borosilicate capillary tube (0.6 mm i.d./ 0.8 mm o.d.; Wilmad Labglass, Inc.). PXRD data were collected on a Bruker AXSD8 Advance A25 Powder X-ray diffractometer (40 kV, 40 mA) using Cu Ka (l = 1.5406 Å) radiation.

Thermogravimetric analysis (TGA)

TGA was measured using a Thermogravimetric Analyzer (TGA), TA Instruments Q500. Typically, ~ 20 mg sample was measured between 25 °C to 900 °C at a heating rate of 10 °C/min under a 40 mL/min nitrogen flow. Prior to any measurement, the samples were dried in an oven at 110 °C to remove water or other solvents.

Electrochemistry

Potentials *versus* Ag/AgCl were converted to the RHE scale using the standard relation $E_{RHE} = E_{Ag/AgCl}^{0} + 0.197 + 0.05916 \times pH$ at room temperature with 0.2 M Na_2SO_4 supporting electrolyte. Note that the light-dark current differences are not affected by the RHE offset. Current densities were normalized by the 0.25 cm^2 exposed working-electrode area.

Illumination measurements were performed with the OAI TriSOL source operated at its nominal 1000 W lamp setting and placed ~ 20 cm from the working electrode. During the measurements, the instrument readouts were 21.3 V and 48.3 A which are lamp power-supply readouts and were not used as an irradiance calibration. Because the irradiance at the working-electrode plane was not measured with a calibrated reference cell, the present data should be interpreted as relative dark/light comparisons under a common illumination condition rather than as absolute mW cm^{-2} photoelectrochemical metrics. Identical illumination condition was used for Cu-BPDC, Zn-BPDC, FTO, and Nafion/FTO.

iR compensation was not applied in this data set, and solution resistance was not measured for this batch. The conclusions therefore, avoid kinetic-slope claims and rely on relative redox features and dark/light current differences collected under the same cell geometry.

Optical Characterization. UV-Vis absorption spectra were collected using a Cary 60 UV-Vis spectrophotometer. Solution measurements were performed on ethanolic dispersions of the MOF-Nafion resin mixtures, with Nafion dissolved in ethanol employed as the blank. The resulting absorbance spectra were used to estimate the optical band gaps by Tauc analysis. Steady-state fluorescence spectra were obtained using a Jobin-Yvon Fluorolog-3 modular spectrofluorometer, model FL-3-11 (HORIBA, Kyoto, Japan), equipped with a 450 W Xe arc lamp excitation source, single-grating monochromators for wavelength selection, and a Hamamatsu R928 photomultiplier tube detector (PMT). Before analysis, all spectra were corrected for instrument response and appropriate blank contributions using standard procedures. Thin films for photoluminescence measurements were prepared by drop-casting a MOF–Nafion resin ink mixture onto FTO substrates, followed by complete drying before analysis. A Nafion-only film deposited on FTO glass was used as the optical blank.

Supporting Figures

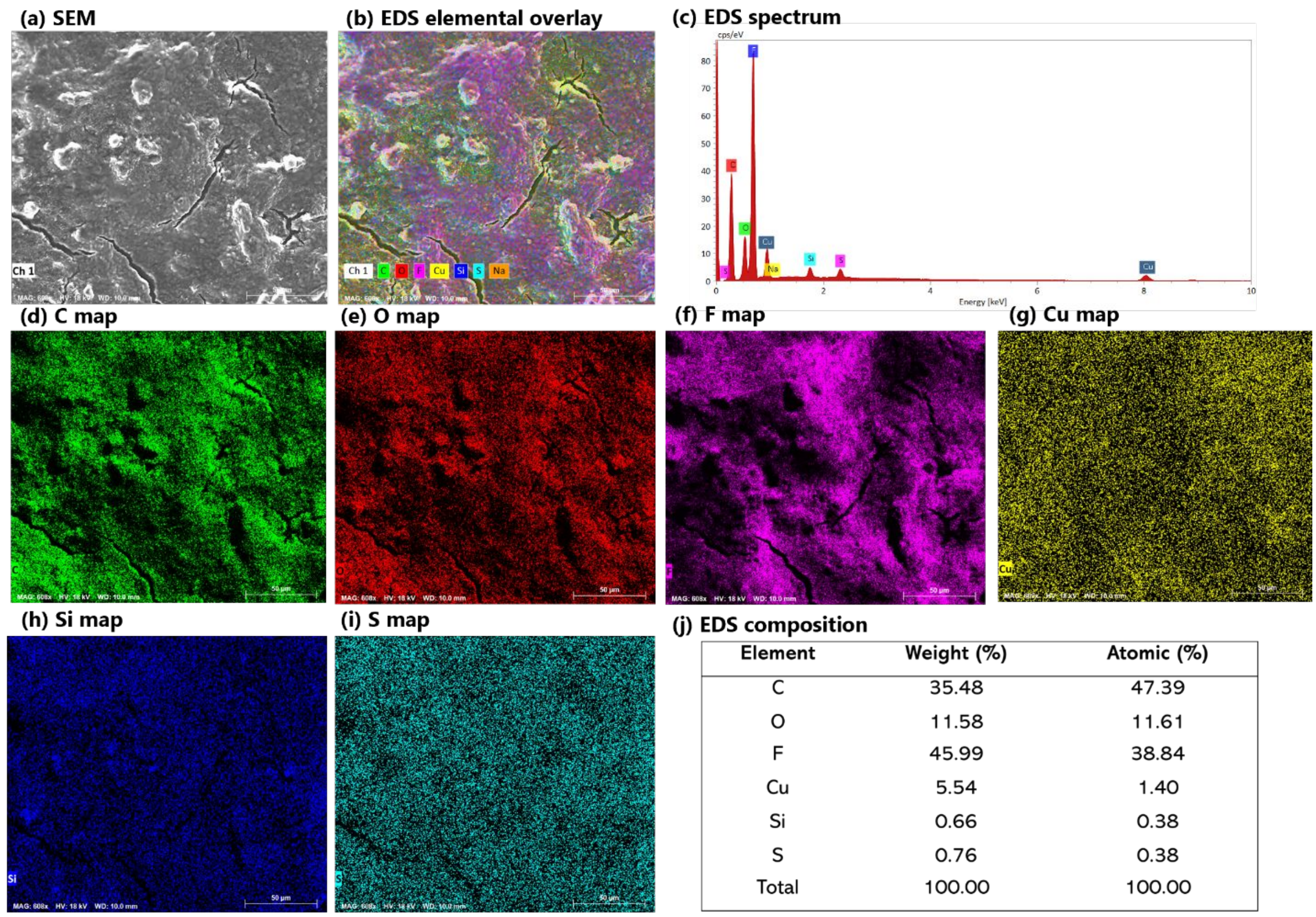

Element	Weight (%)	Atomic (%)
C	35.48	47.39
O	11.58	11.61
F	45.99	38.84
Cu	5.54	1.40
Si	0.66	0.38
S	0.76	0.38
Total	100.00	100.00

Figure S1. SEM–EDS characterization of the Cu-BPDC-coated FTO electrode before photoelectrochemical testing. (a) SEM micrograph, (b) EDS elemental overlay, (c) EDS spectrum, elemental maps of (d) C, (e) O, (f) F, (g) Cu, (h) Si, (i) S, and (j) quantitative EDS composition. The broadly distributed Cu signal confirms the presence of Cu-containing material throughout the analyzed coating, while F and S are consistent with contributions from the Nafion-containing binder.

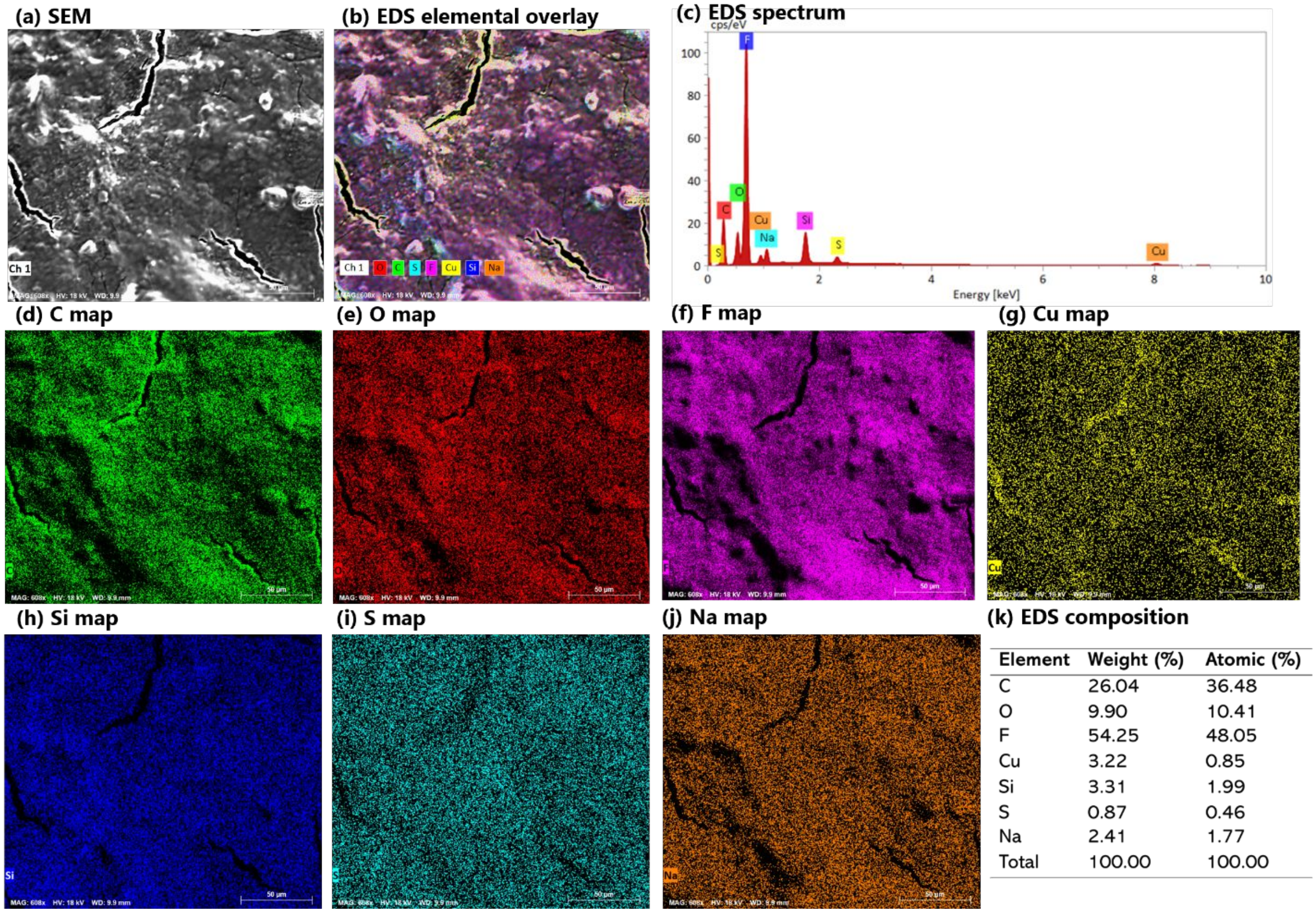

Element	Weight (%)	Atomic (%)
C	26.04	36.48
O	9.90	10.41
F	54.25	48.05
Cu	3.22	0.85
Si	3.31	1.99
S	0.87	0.46
Na	2.41	1.77
Total	100.00	100.00

Figure S2. SEM–EDS characterization of the Cu-BPDC-coated FTO electrode after photoelectrochemical testing. (a) SEM micrograph, (b) EDS elemental overlay, (c) EDS spectrum, elemental maps of (d) C, (e) O, (f) F, (g) Cu, (h) Si, (i) S, (j) Na, and (k) quantitative EDS composition. Cu remains distributed across the electrode following testing, although the reduced Cu fraction and increased Si contribution indicate post-experiment compositional redistribution and/or greater exposure of the underlying substrate. Na in the post-experiment sample may reflect residual sodium-containing electrolyte.

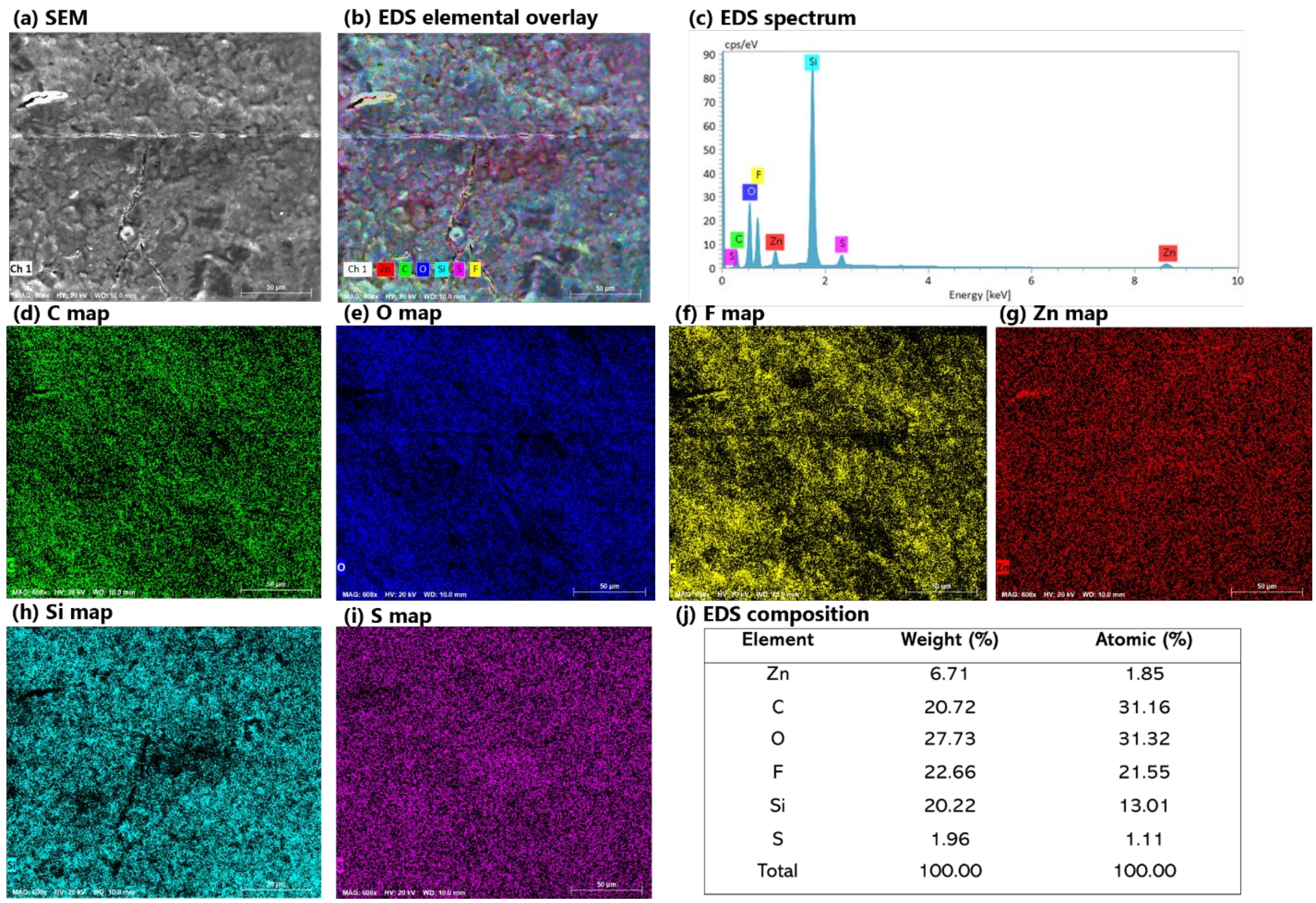

Element	Weight (%)	Atomic (%)
Zn	6.71	1.85
C	20.72	31.16
O	27.73	31.32
F	22.66	21.55
Si	20.22	13.01
S	1.96	1.11
Total	100.00	100.00

Figure S3. SEM–EDS characterization of the Zn-BPDC-coated FTO electrode before photoelectrochemical testing. (a) SEM micrograph, (b) EDS elemental overlay, (c) EDS spectrum, elemental maps of (d) C, (e) O, (f) F, (g) Zn, (h) Si, (i) S, and (j) quantitative EDS composition. Zn is widely distributed throughout the analyzed region, whereas the strong Si signal indicates a substantial contribution from the underlying substrate to the EDS interaction volume.

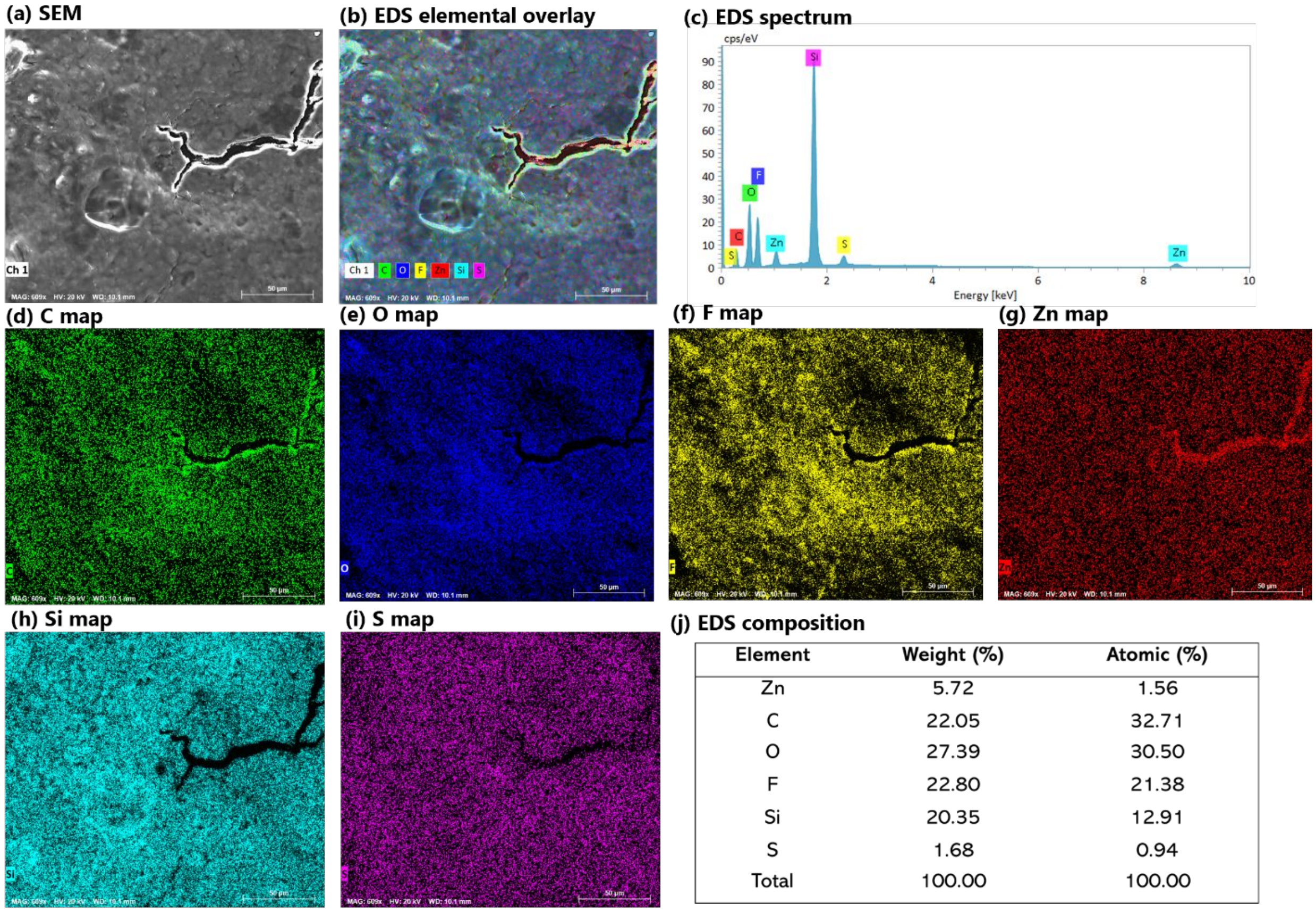

Element	Weight (%)	Atomic (%)
Zn	5.72	1.56
C	22.05	32.71
O	27.39	30.50
F	22.80	21.38
Si	20.35	12.91
S	1.68	0.94
Total	100.00	100.00

Figure S4. SEM–EDS characterization of the Zn-BPDC-coated FTO electrode after photoelectrochemical testing. (a) SEM micrograph, (b) EDS elemental overlay, (c) EDS spectrum, elemental maps of (d) C, (e) O, (f) F, (g) Zn, (h) Si, (i) S, and (j) quantitative EDS composition. The persistence of broadly distributed Zn, together with only modest changes in elemental composition, indicates substantial retention of the Zn-containing coating's composition following testing.

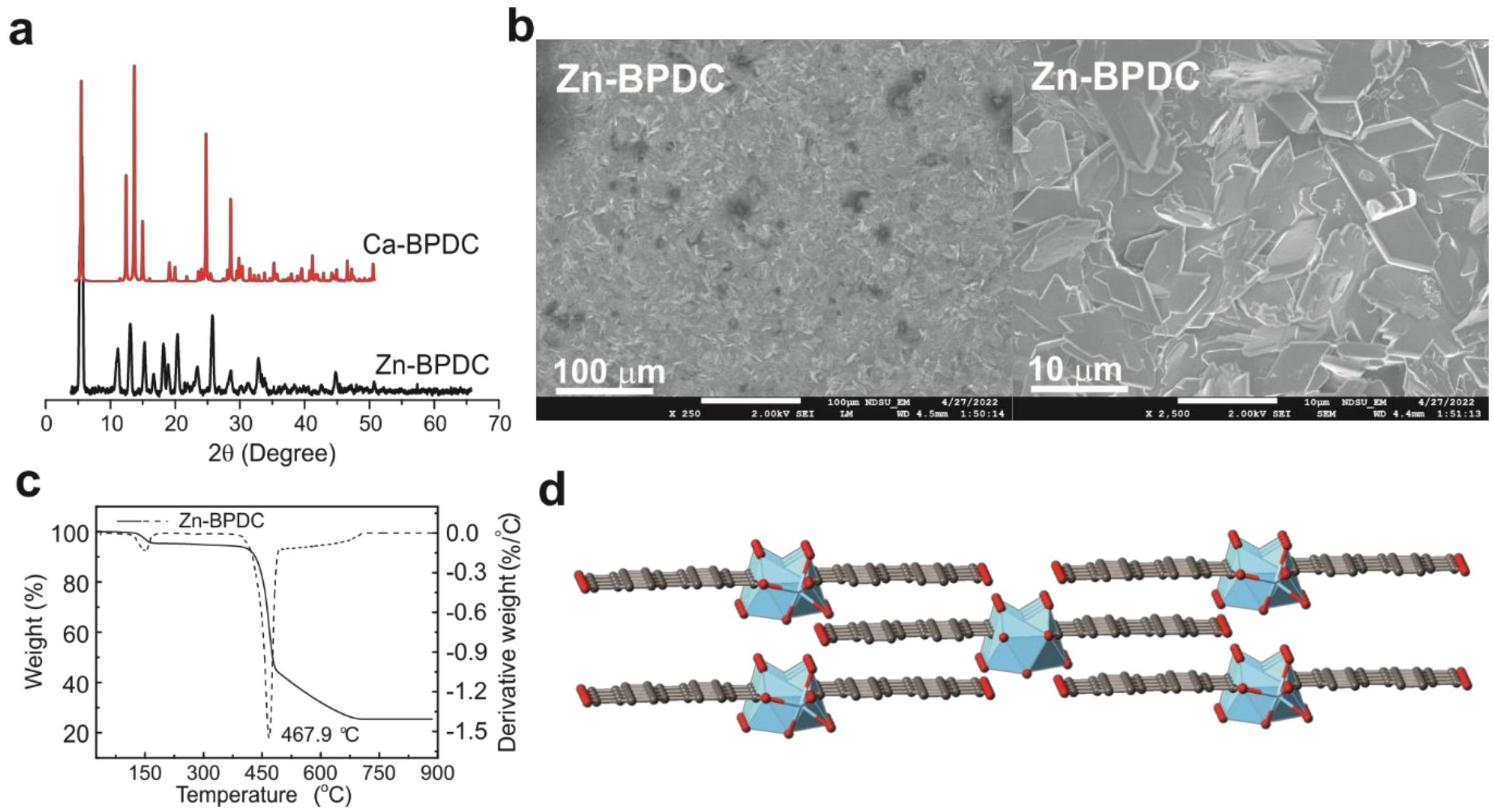

Figure S5. Additional characterization of Zn(II)-BPDC via PXRD (a), SEM at two scales (b), and TGA (c). A proposed structure based on simulation of the PXRD is displayed in (d), consistent with our recent finding.[2]

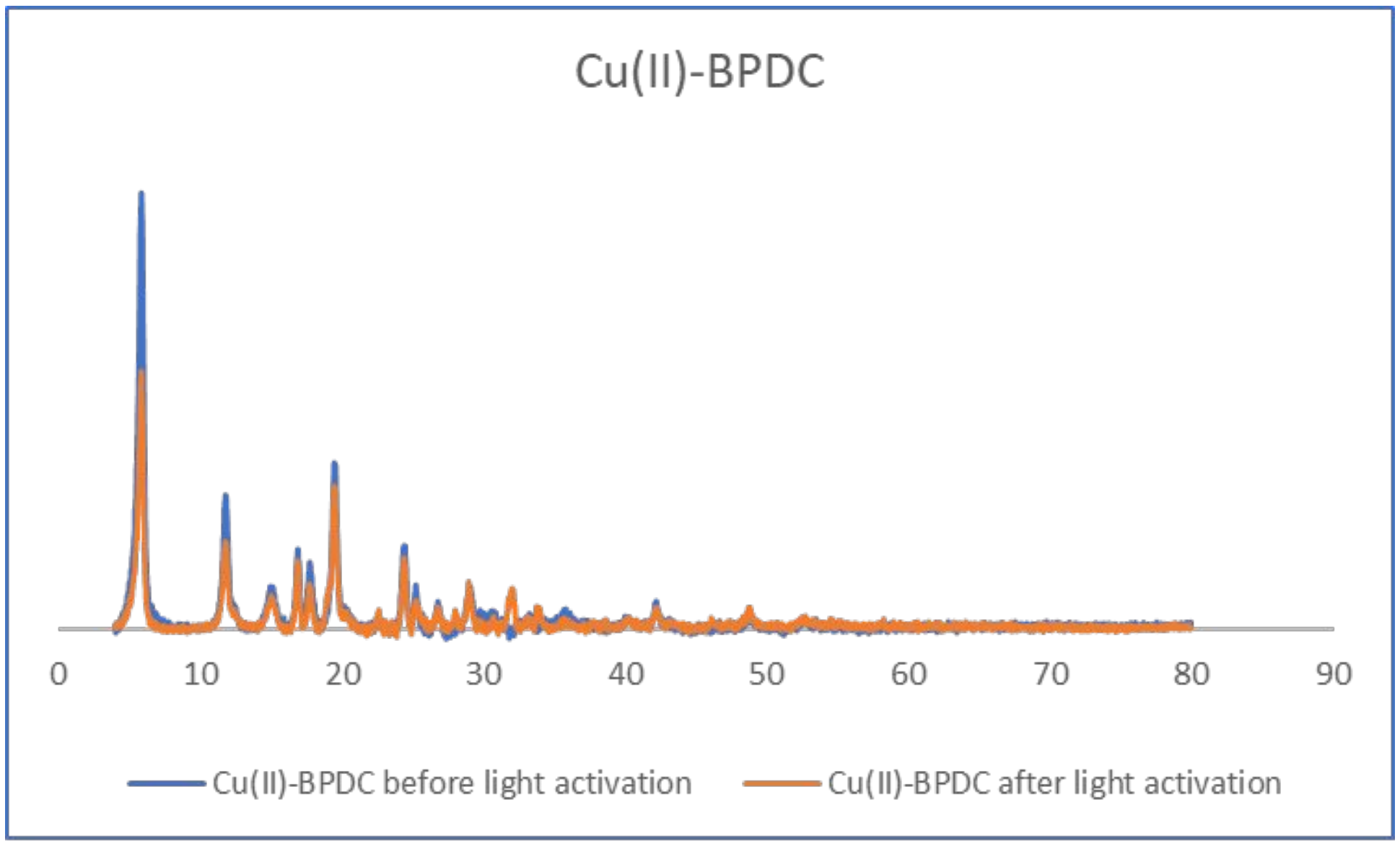

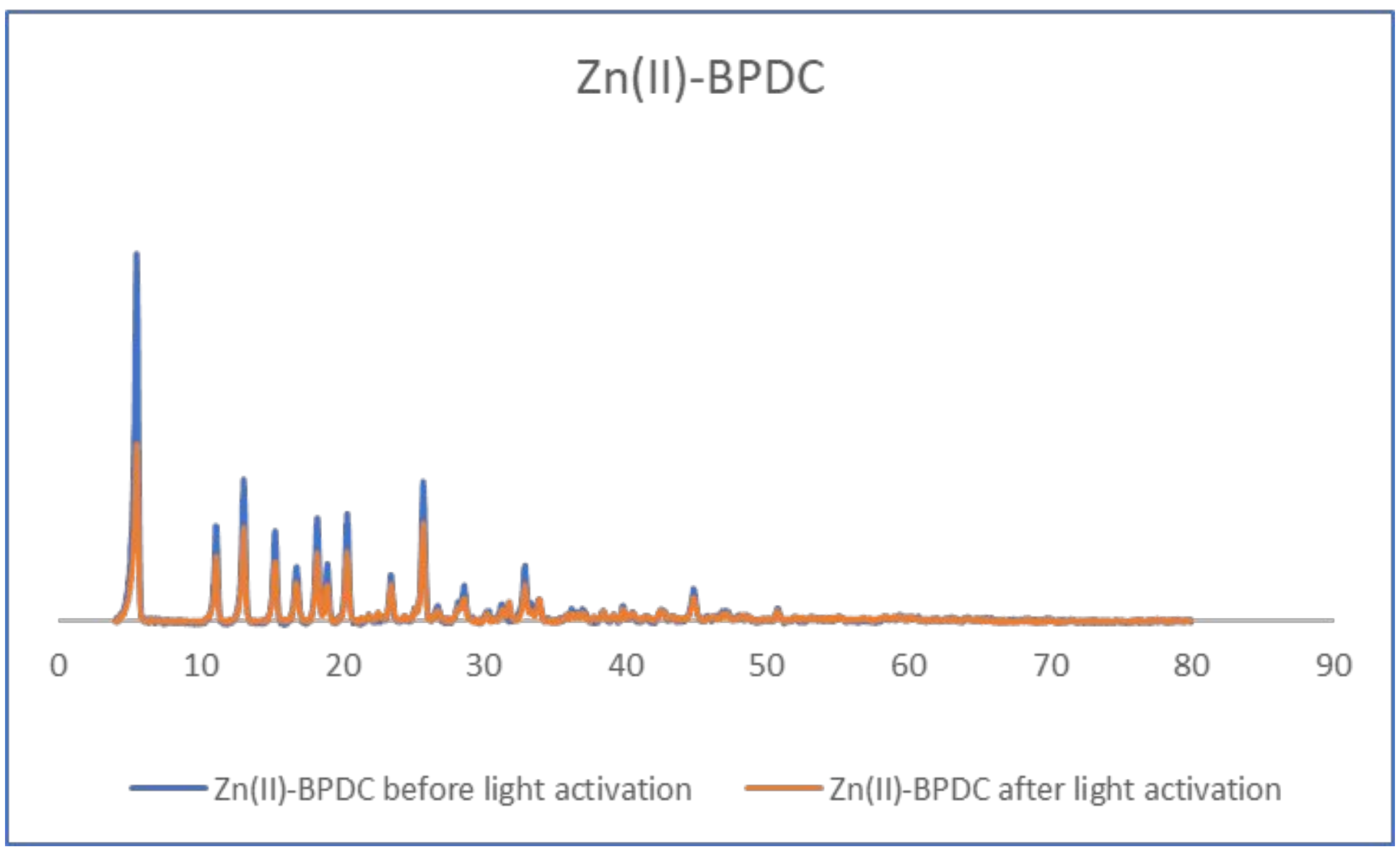

Figure S6. Overlapping of PXRD patterns of involved MOFs in this work before and after light exposure.

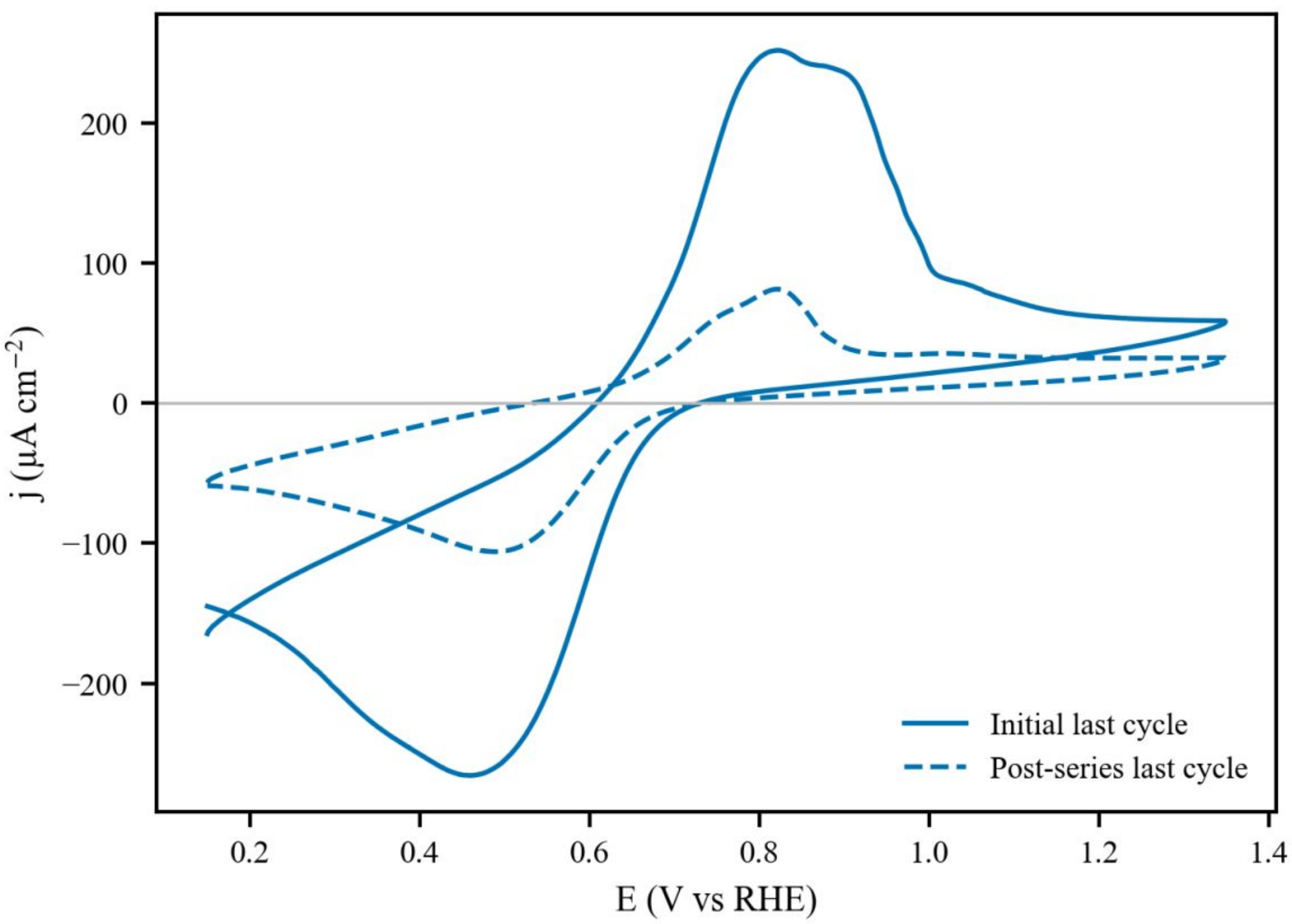

Figure S7. Last-cycle comparison of CV before and after the scan-rate series for Cu-BPDC (100 mV s^{-1}).

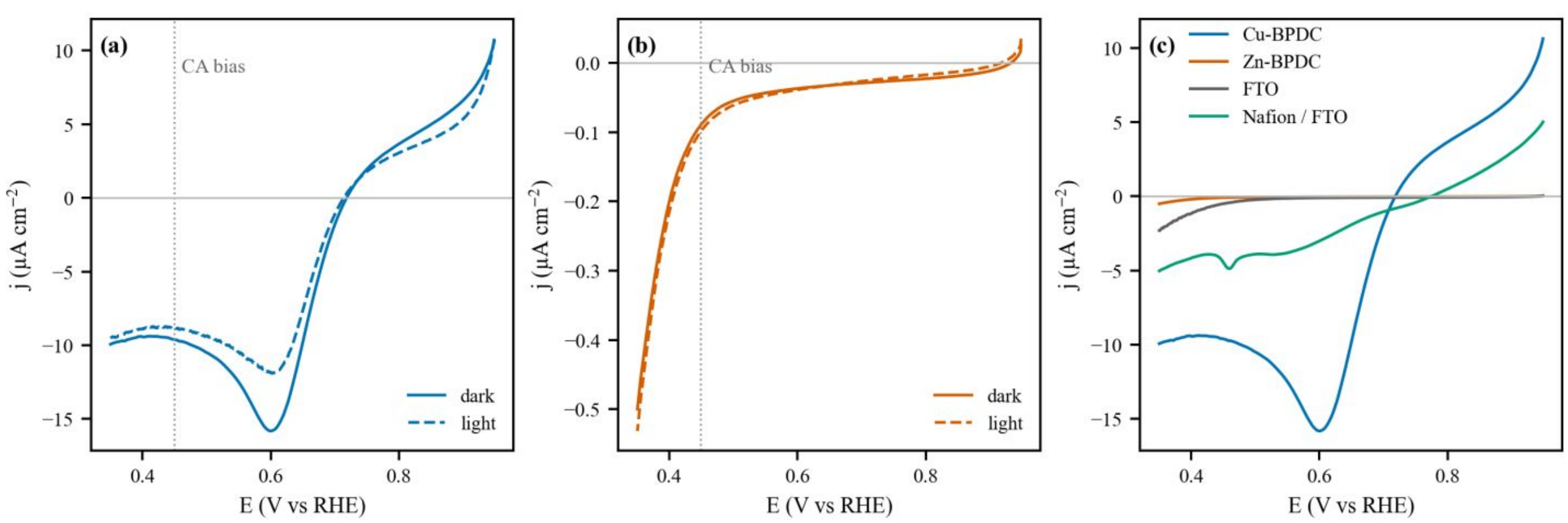

Figure S8: Dark/light linear sweep voltammetry (LSV) traces and substrate baselines. (a) Cu-BPDC dark/light LSV traces. (b) Zn-BPDC dark/light LSV traces. The vertical guide in each figure marks the CA bias at +0.449 V versus RHE. (c) Dark-current comparison across Cu-BPDC, Zn-BPDC, FTO, and Nafion/FTO.

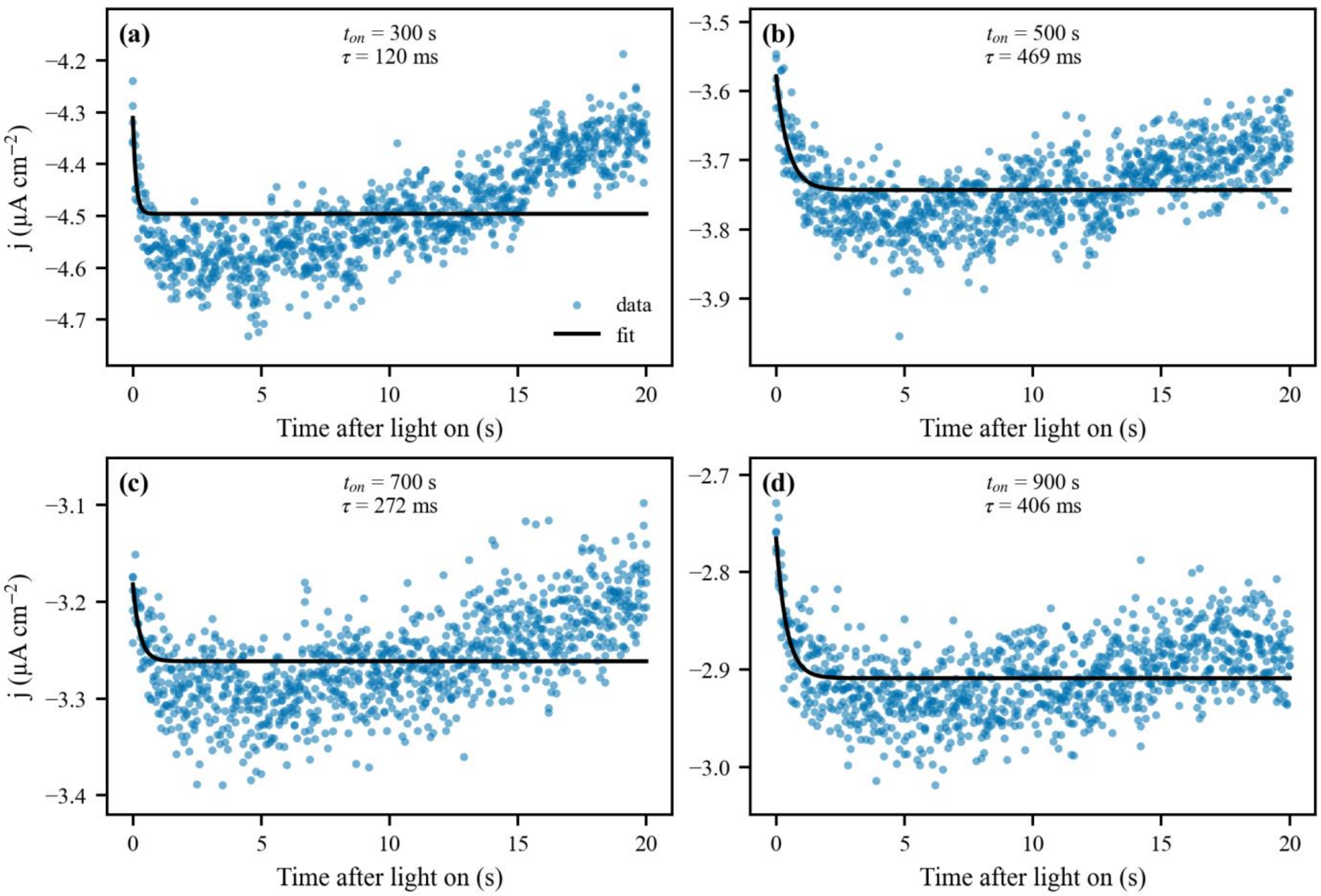

Figure S9: Light-on transient fits for Cu-BPDC during the chopped-light CA measurement for cycles 2-5.

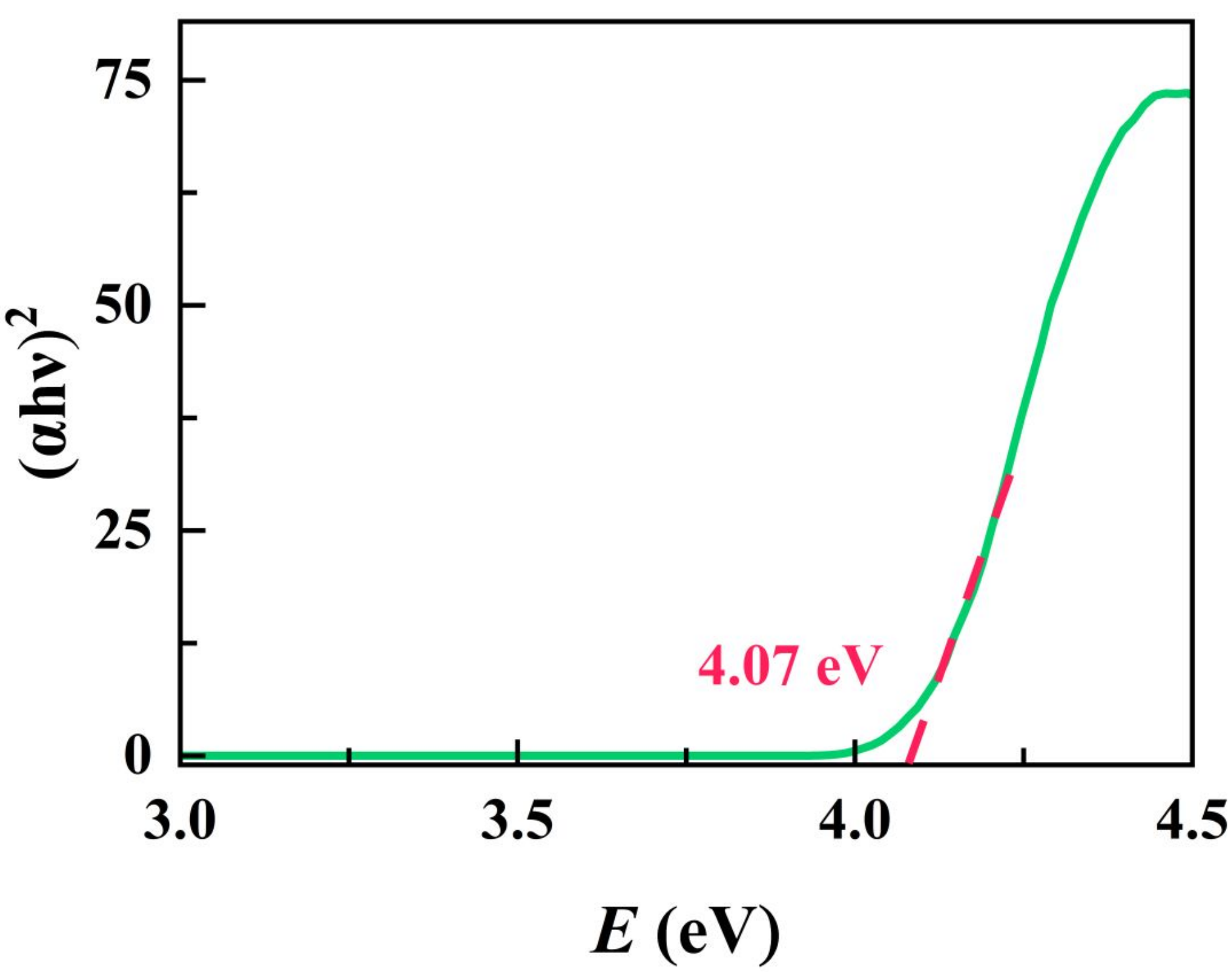

Figure S10. Direct-transition Tauc plot of the free Na_2BPDC linker, used to estimate its apparent optical band-gap energy, *E*g. The plot of $(\alpha h\nu)^2$ *versus* photon energy (hν) is shown, where α is the absorption coefficient, yielding a value of 4.07 eV.

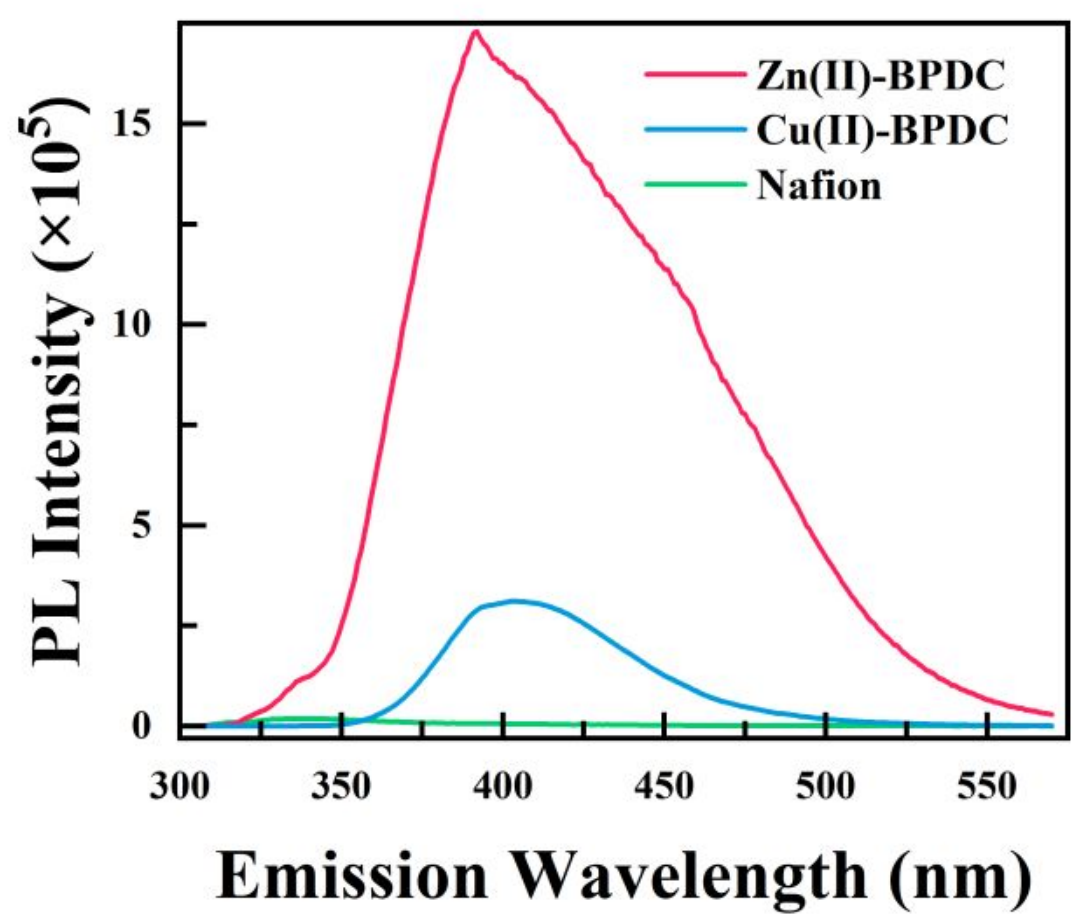

Figure S11. Photoluminescence spectra of Zn-BPDC and Cu-BPDC MOF solid-state thin films deposited on FTO glass.

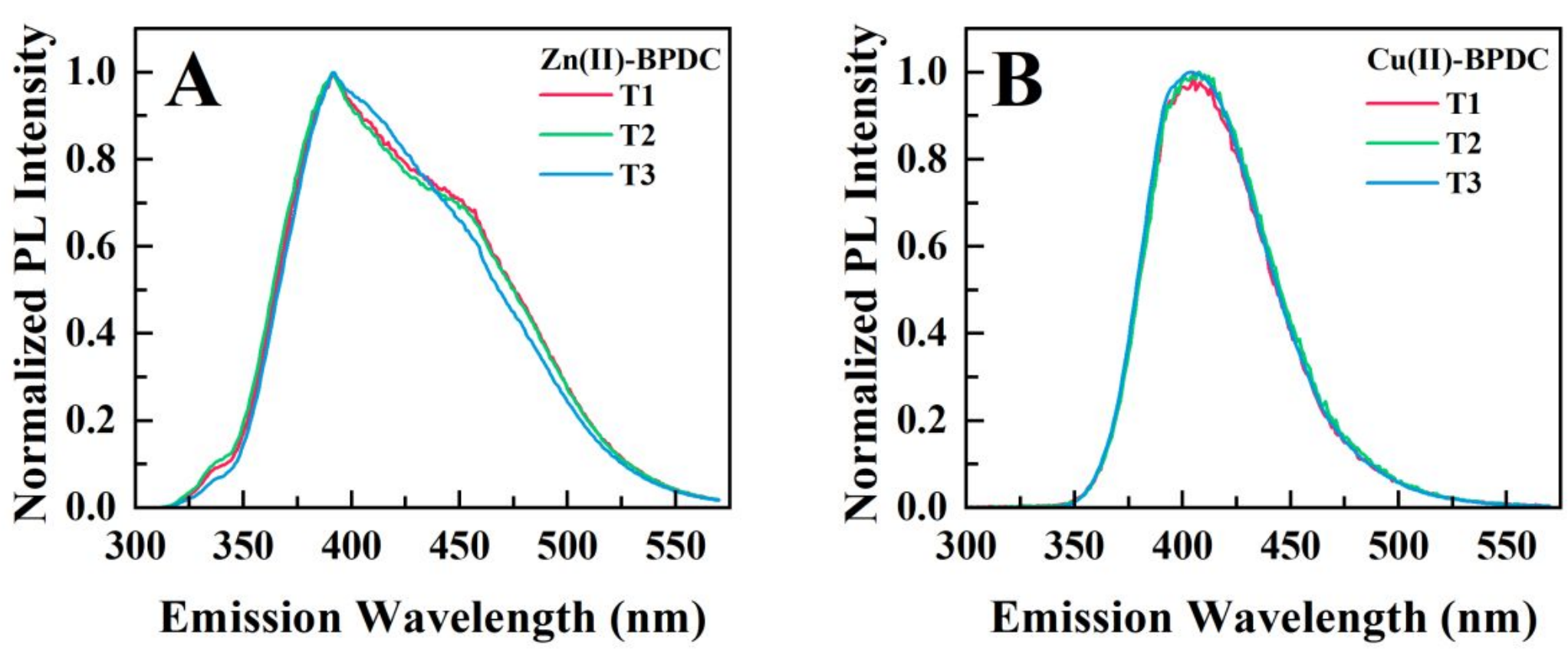

Figure S12. Normalized photoluminescence spectra of solid-state thin films of (A) Zn-BPDC and (B) Cu-BPDC MOFs deposited on FTO glass over 3 trials (T1-T3), demonstrating sample-to-sample spectral consistency.

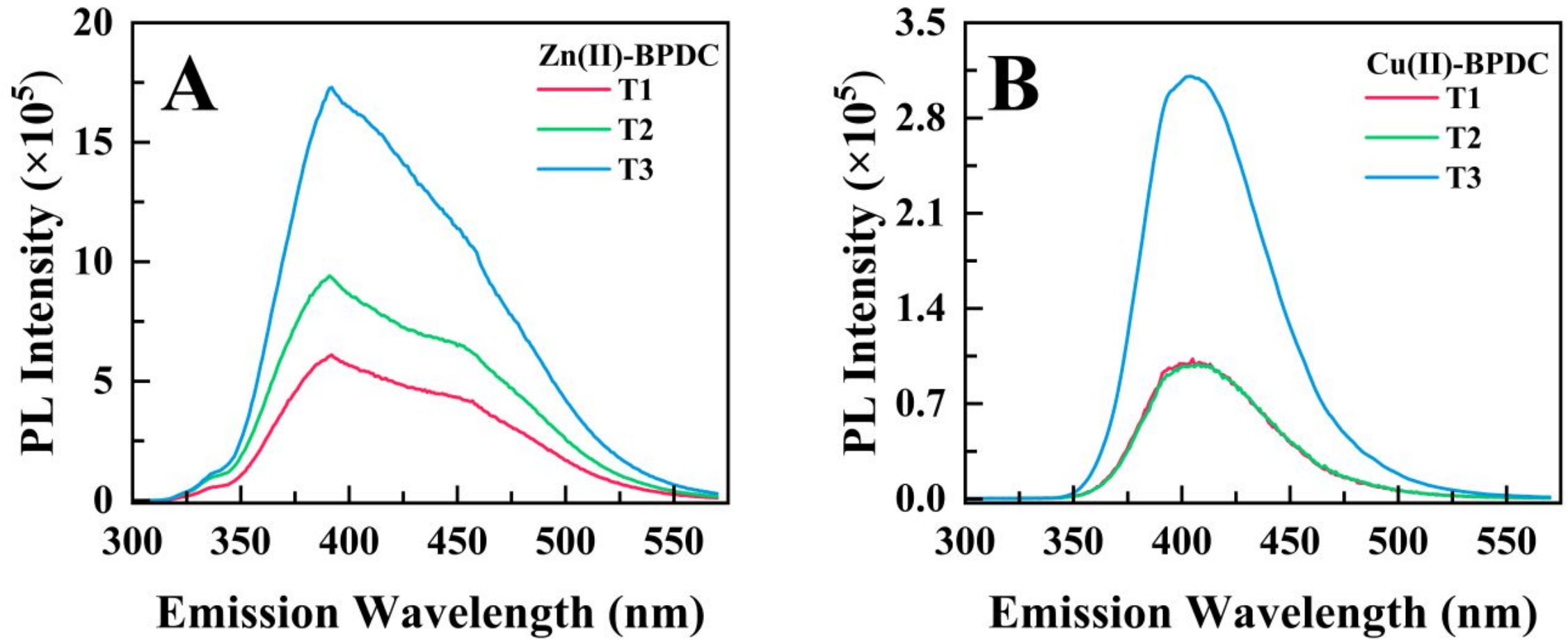

Figure S13. Solid-state PL spectra of (A) Zn(II)-BPDC and (B) Cu(II)-BPDC MOF thin films on FTO glass. The films were fabricated by drop-casting a MOF–Nafion resin ink mixture onto fluorine-doped tin oxide (FTO) substrates and drying before analysis.

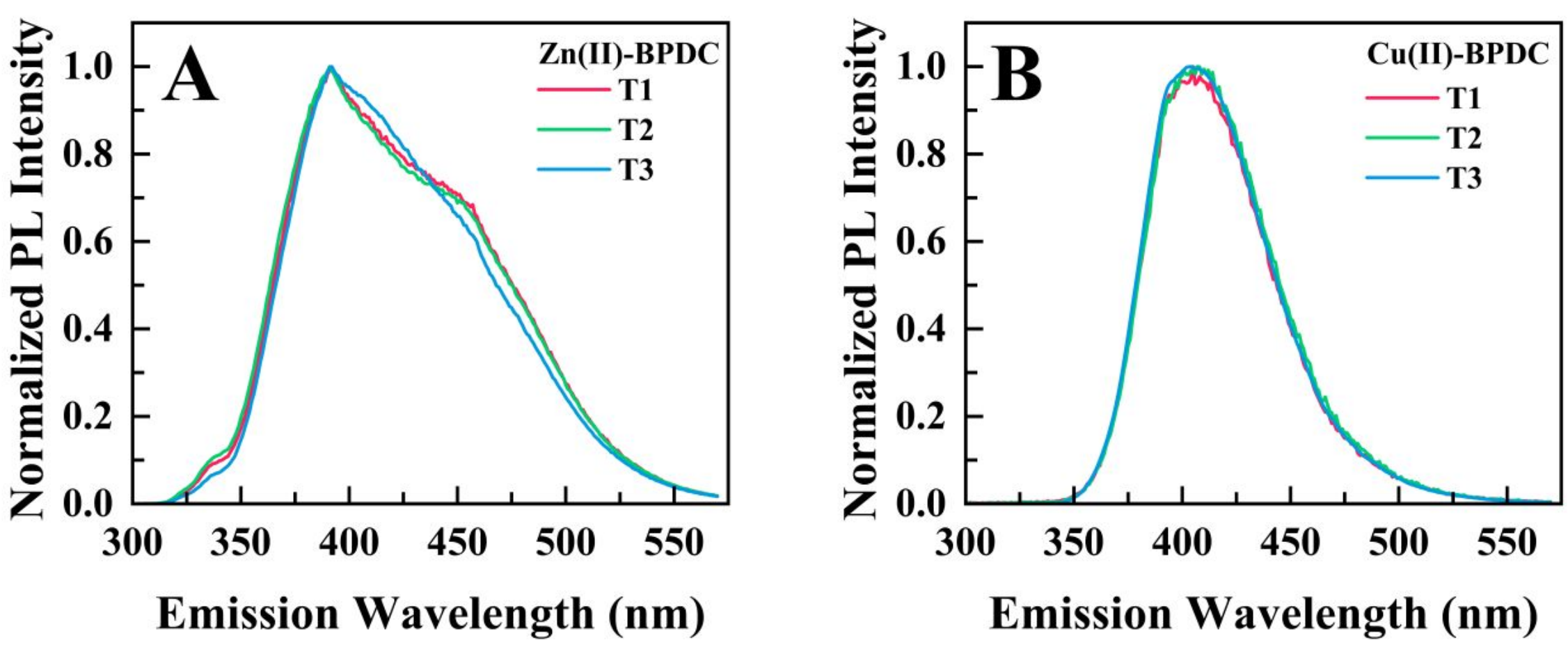

Figure S14. Solid-state normalized PL spectra of (A) Zn(II)-BPDC and (B) Cu(II)-BPDC MOF thin films on FTO glass. The films were fabricated by drop-casting a MOF–Nafion resin ink mixture onto fluorine-doped tin oxide (FTO) substrates and drying before analysis.

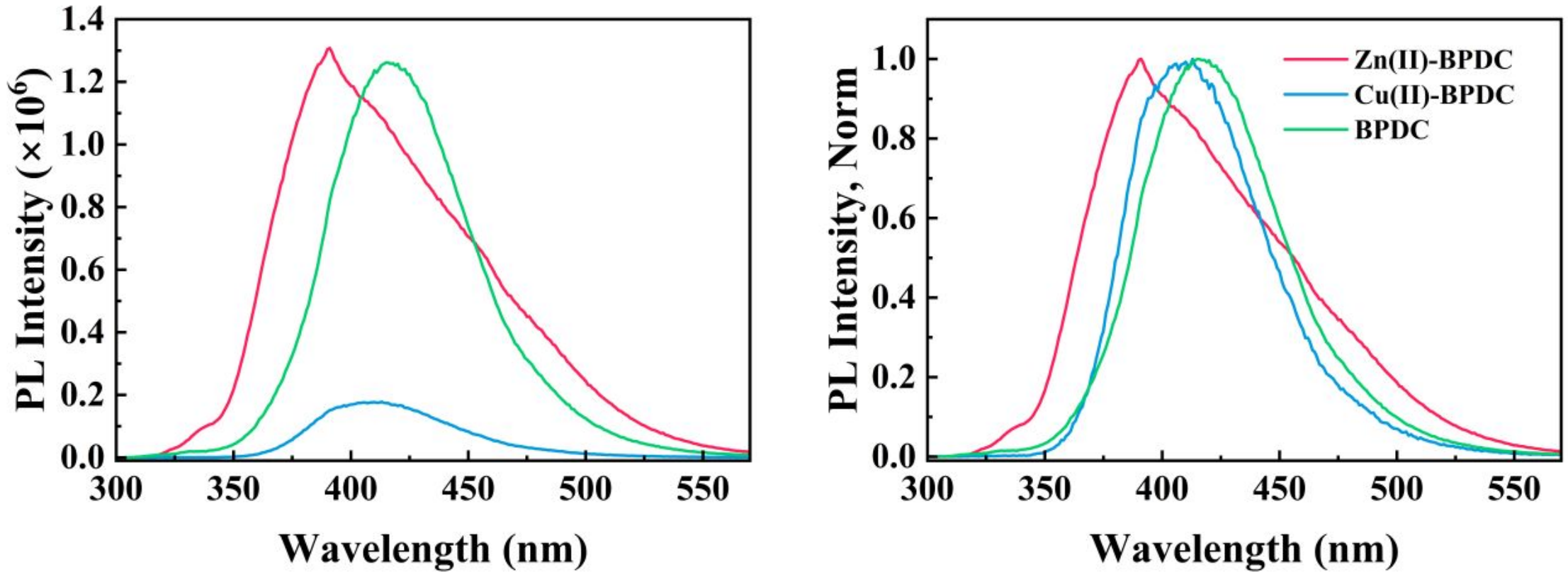

Figure S15. Steady-state photoluminescence spectra of BPDC, Zn(II)-BPDC, and Cu(II)-BPDC. (Left) As-measured PL spectra, with emission maxima at 416, 392, and 414 nm, respectively. (Right) Normalized PL spectra, showing the blue-shifted emission maximum and broadened red tail of Zn(II)-BPDC relative to the free linker.

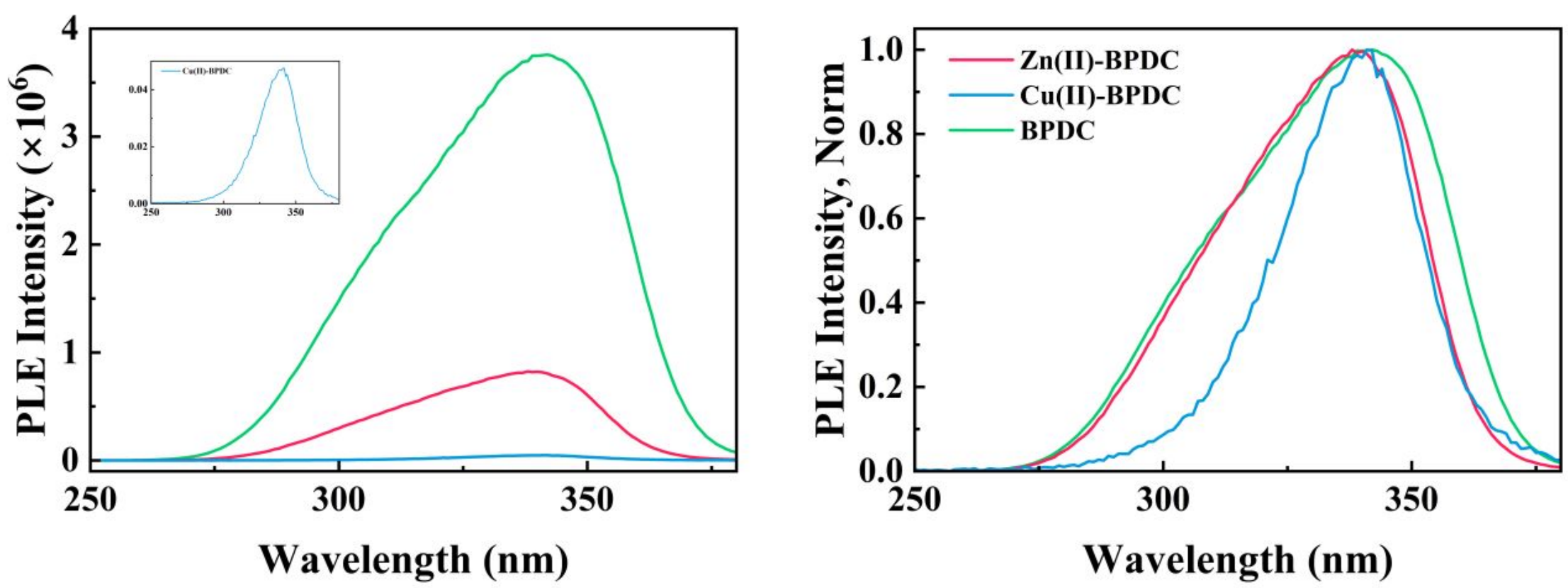

Figure S16. Photoluminescence excitation (PLE) spectra of BPDC, Zn(II)-BPDC, and Cu(II)-BPDC monitored at their respective emission maxima. (Left) As-measured PLE spectra; the inset shows the Cu(II)-BPDC spectrum on an expanded intensity scale. (Right) Normalized PLE spectra, showing that all three materials exhibit closely similar excitation profiles with maxima at ~340 nm, consistent with a BPDC-centered origin of the emissive excited state in both frameworks.

Supporting Tables

Table S1: Last-cycle CV cathodic and anodic reference values.

Material	Scan rate, ν ($mV\ s^{-1}$)	Cathodic E (V vs RHE)	Cathodic current-density magnitude ($\mu A\ cm^{-2}$)	Anodic E (V vs RHE)	Anodic current-density magnitude ($\mu A\ cm^{-2}$)
Cu-BPDC	25	+0.517	131.7	+0.743	130.1
Cu-BPDC	100	+0.476	154.7	+0.776	202.9
Cu-BPDC	200	+0.456	191.7	+0.817	316.1
Zn-BPDC	25	+0.150	11.4	—	—
Zn-BPDC	50	+0.150	11.0	—	—
Zn-BPDC	100	+0.150	11.6	—	—
Zn-BPDC	200	+0.151	13.1	—	—

Table S2. Fluorescence Decay Parameters and Intensity-Weighted Average Lifetimes of Free BPDC, Zn(II)-BPDC, and Cu(II)-BPDC Obtained from Triexponential Fits of TCSPC Decays [a]

Sample	τ_1 (ns)	B_1 (%)	τ_2 (ns)	B_2 (%)	τ_3 (ns)	B_3 (%)	$\langle\tau\rangle$ (ns)[b]	χ^2
**BPDC**	13.19	25.51	42.25	63.98	2.62	10.51	38.7	1.35
**Zn(II)-BPDC**	1.39	38.66	3.50	48.96	13.22	12.38	7.3	1.22
**Cu(II)-BPDC**	3.56	47.19	1.26	33.05	13.57	19.76	9.2	1.17

[a] Decays were fit to a triexponential model, $I(t) = \sum_i B_i e^{(-\frac{t}{\tau_i})}$; B_i denotes the relative amplitude of the ith component.

[b] Intensity-weighted average lifetime, $\langle\tau\rangle = \sum_i B_i \tau_i^2 / \sum_i B_i \tau_i$

Table S3: LSV dark current densities at selected potentials.

E (V vs RHE)	Cu-BPDC dark (μA cm⁻²)	Zn-BPDC dark (μA cm⁻²)	FTO dark (μA cm⁻²)	Nafion/FTO dark (μA cm⁻²)
+0.449	−9.60	−0.09	−0.50	−4.39
+0.400	−9.43	−0.20	−1.17	−4.16
+0.350	−9.93	−0.50	−2.31	−5.03

Table S4: Chopped-light CA statistics from cycles 2-5 within a single CA measurement. The cathodic response is reported as $-\Delta j$, where $\Delta j = j_{light} - j_{dark}$; positive values therefore indicate a more cathodic current under illumination.

Material	CA bias (V vs RHE)	Cathodic response, −Δj (μA cm⁻²)	SE (μA cm⁻²)
Cu-BPDC	+0.449	+0.4477	0.0094
Zn-BPDC	+0.449	+0.0014	0.0001
FTO	+0.449	−0.0044	0.0022
Nafion/FTO	+0.449	−0.0262	0.0084

Supporting References

(1) Dipti, T. T.; Hasan, M. Z.; Armstrong, Z.; Quadir, M.; Liu, L.; Wan, C.; Baker, G. A.; Feng, L.; MacRae, A.; Yang, Z. Water as an Eco-friendly Liquid-Assisted Grinding Agent (ecoLAGent) to Expand the Metal–Organic Framework "Library" for Green Enzyme Encapsulation. *ACS Appl. Mater. Interfaces* **2025**, *17* (39), 55615-55627. DOI: 10.1021/acsami.5c14214.

(2) Jordahl, D.; Armstrong, Z.; Li, Q.; Gao, R.; Liu, W.; Johnson, K.; Brown, W.; Scheiwiller, A.; Feng, L.; Ugrinov, A.; et al. Expanding the library of Metal-Organic Frameworks (MOFs) for enzyme biomineralization. *ACS Appl. Mater. Interfaces* **2022**, *14* (46), 51619-51629.
